# Volatile Organic Compounds on Rhodes Island, Greece: Implications for Outdoor and Indoor Human Exposure

**DOI:** 10.3390/toxics12070486

**Published:** 2024-07-02

**Authors:** Athanasios Besis, Dimitrios Margaritis, Constantini Samara, Evangelos Bekiaris

**Affiliations:** 1 Centre for Research and Technology Hellas (CERTH)/Hellenic Institute of Transport (HIT), GR-57001 Thessaloniki, Greece; dmarg@certh.gr (D.M.); abek@certh.gr (E.B.); 2 Environmental Pollution Control Laboratory, Department of Chemistry, Aristotle University of Thessaloniki, GR-54124 Thessaloniki, Greece; csamara@chem.auth.gr

**Keywords:** VOCs, Rhodes, indoor/outdoor, positive matrix factorization (PMF), human exposure, carcinogenic/non-carcinogenic risk

## Abstract

Volatile organic compounds (VOC) are considered a class of pollutants with a significant presence in indoor and outdoor air and serious health effects. The aim of this study was to measure and evaluate the levels of outdoor and indoor VOCs at selected sites on Rhodes Island, Greece, during the cold and warm periods of 2023. Spatial and seasonal variations were evaluated; moreover, cancer and non-cancer inhalation risks were assessed. For this purpose, simultaneous indoor-outdoor air sampling was carried out on the island of Rhodes. VOCs were determined by Thermal Desorption—Gas Chromatography/Mass Spectroscopy (TD-GC/MS). Fifty-six VOCs with frequencies ≥ 50% were further considered. VOC concentrations (∑_56_VOCs) at all sites were found to be higher in the warm period. In the warm and cold sampling periods, the highest concentrations were found at the port of Rhodes City, while total VOC concentrations were dominated by alkanes. The Positive Matrix Factorization (PMF) model was applied to identify the VOC emission sources. Non-cancer and cancer risks for adults were within the safe levels.

## 1. Introduction

Human processes are considered the largest sources of volatile-organic-compound (VOC) emissions in the atmosphere of urban areas. They mainly include emissions from mobile sources, such as cars, airplanes, ships, etc., [1,2] and from point sources, such as oil-refining industries, chemical plants, landfills, waste-treatment plants, power plants, etc. [3,4,5,6,7,8,9,10]. In addition, VOCs can be transferred from sources located hundreds of kilometers away [11,12]. The determination of VOC emissions from various sources is essential to further our understanding of their impacts on urban air quality and public health [13]. Several studies have focused on seasonal variations and the correlation of VOC concentrations with meteorological conditions [13,14,15,16]. The ambient temperature has a significant influence on VOC emissions; however, the link between temperature and anthropogenic VOC sources is still not well understood because the ambient VOCs come from multiple sources [13] and are also important precursors of secondary air pollutants and secondary organic aerosols (SOA) in photochemical processes [17,18].

VOCs are also detected in indoor environments, such as homes, schools, and workplaces [19,20,21,22,23]. People spend between 80 and 95% of their time indoors, so the quality of indoor air plays a significant role in total human exposure to air pollutants [7]. Important sources of VOCs in indoor air, beyond the transportation of outdoor air [24], are smoking, household cleaning, cooking, electronic office equipment, building materials, furniture, other static contents, etc. [25,26]. VOCs also form from the ozonolysis of human skin oils [27,28,29,30].

The ratios of indoor and outdoor (I/O) VOC levels can be determined using paired measurements of indoor and outdoor air [31]. The I/O ratio is generally used to infer penetration to indoor environments and indoor sources. Significant indoor sources are indicated by an I/O ratio greater than 1, whereas additional sources from external air or the removal of VOCs indoors are suggested by a low I/O ratio [32,33,34,35].

VOCs are lipophilic compounds and, therefore, accumulate in the fatty tissues of the human body. VOCs have been found to be responsible for various health problems in humans [36]. It is possible for them to enter the human body through inhalation and skin absorption, while health effects can be due to exposure to each individual compound or to all VOCs [37]. As a result of gas-phase oxidation reactions, partitioning between the gas and condensed phases, multiphase reactions on indoor surfaces, or airborne particles, many VOCs are changed into semi-volatile species during transportation from outside to indoors. These transformations then affect the concentrations and compositions of indoor pollutants [38]. The overall balance of human exposure to VOCs is, therefore, a blend of indoor- and outdoor-inhaled air [39].

The aim of this study was to measure and evaluate the levels of VOCs on Rhodes Island, with a focus on the city of Rhodes, in outdoor and indoor air during the cold and warm periods of the year 2023. To the best of our knowledge, this is the first study that reports VOC levels in indoor and outdoor air on Rhodes Island. There are only a few studies reporting VOC concentrations on islands (Nisyros Island at a western Mediterranean remote background site [40]; Corsica [41]; Taiwan [42]; and Reunion Island in the Indian Ocean [43]). Islands need to be connected to the outside world via sea or air. As a result, islands have port infrastructures and, in some cases, airports. Ports are major sources of air pollution [44]. Emissions are generated by the maneuvering ships and the activity at the dock, such as the incessant movement of vehicles and cargo vehicles, and also while the ships are at berth since not all types of ships switch off the main engines [45,46]. Ship emissions in harbors can have a significant impact on local air quality, population exposure, and, therefore, human health in urban areas [47]. Airports are also significant contributors to air pollution, with the main sources being airplanes and ground-support equipment that often run on diesel and the rental-car fleet [48].

VOC sampling was carried out at six sites during the cold and warm periods of 2023. A total of 56 VOCs were identified, including 23 aromatics, 12 aldehydes, 2 ketones, 8 alkanes, 6 alkenes, and 5 acids. The indoor-outdoor ratios were calculated to get an inference regarding the sources of VOC exposure and the impact of outdoor-related air pollution on the indoor environment. Also, PMF was applied to the VOC measurements to gain insights about their sources.

## 2. Materials and Methods

### 2.1. Site and Sampling Description

Rhodes Island, located in the southeastern Aegean Archipelagos, is the largest of the Dodecanese islands of Greece (Latitude: 36°26′26″ N; Longitude: 28°13′21″ E). The principal town on the island and seat of the municipality is Rhodes. The island of Rhodes has a total area of ~1400 km^2^. In 2022, the island had a population of 125,113 people. The city of Rhodes is located at the northern tip of the island, with a population of ~50,636 inhabitants.

Rhodes Island attracts more than two million non-resident visitors per year, which is approximately 10% of the Greek tourist product [49]. The medieval city of Rhodes is currently a UNESCO World Heritage Site, enhancing the island’s tourist value. Therefore, it can be assumed that the Island of Rhodes is one of the most popular tourist destinations worldwide [49]. Rhodes has an Eastern Mediterranean climate, with mild winters and hot summers. Lindos is a traditional settlement on the island of Rhodes and, according to the Hellenic National Meteorological Service, for the period 2010–2019, was the warmest area in Greece (mean annual temperature of 21.9 °C). The island has two airports, but only Diagoras Airport (southwest of Rhodes City) is public. The number of international air arrivals to Rhodes in 2022 was ~2.5 million. Rhodes also has five ports, three of them in Rhodes City.

**Figure 1:**
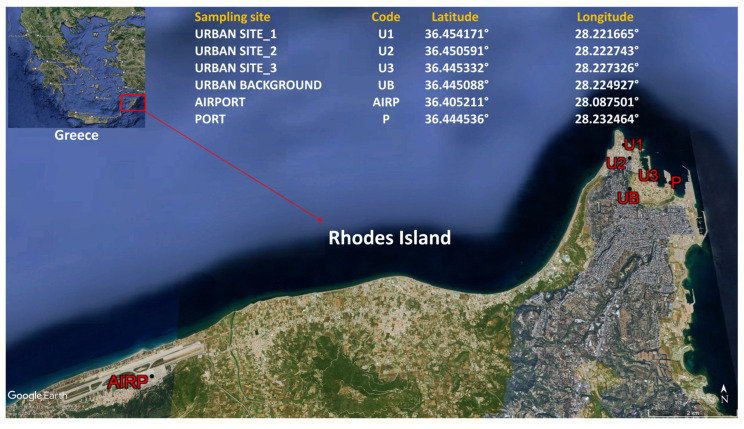
Map of the Island of Rhodes and the sampling sites in the city of Rhodes. U1: Urban site 1 (Museum) (indoor/outdoor sampling); U2: Urban site 2 (Hotel) (indoor/outdoor sampling); U3: Urban site 3 (Offices) (indoor/outdoor sampling); UB: Urban background (outdoor sampling); AIR: Airport (indoor/outdoor sampling); P: Port (indoor/outdoor sampling).

Five sampling sites were selected in the city of Rhodes, including three urban sites (U1, U2, U3), one urban background site (UB), and the port (P), while a sixth site was selected in Diagoras Airport, located ~13 km NW of the city (Figure 1). The selection of sampling sites was based mainly on the following factors: absence of nearby emission sources, accessibility, and representativeness of a large part of the city. Excepting UB, at all other five sites (U1, U2, U3, P, and AIRP), outdoor sampling of VOCs was accompanied by concurrent indoor sampling. Data for meteorological conditions prevailing during VOC samplings (ambient temperature, relative humidity, and wind direction/speed) (Table S1a) were obtained from the meteorological station at the port sampling site and were considered the same for all sampling sites. The influential factors in the sampled indoor sites (type, size, and potential sources) are available in the Supplementary Materials (Table S1b). All indoor sites used air conditioning systems except the offices in the port, which were ventilated naturally. There were no smokers or other sources, such as fireplaces, in the indoor sampling sites.

One indoor and outdoor air sample was collected per sampling site and per sampling day between 09:00 a.m. and 01:00 p.m. during the cold (March) and the warm periods (July) of 2023. In total, sixty-six (66) indoor and outdoor samples (30 and 36 samples, respectively) of air were collected. Sampling was carried out according to Besis et al. [2,9,10] using portable pumps Gillian GILAIR-Plus Personal Air Sampling Pump, Sensidyne, St. Petersburg, FL, USA (1–5000 cm^3^ min^−1^) and adsorption-thermal desorption columns (Markes International Limited, Llantrisant, UK). Inert-coated stainless-steel tubes were used, packed with a carefully optimized combination of weak and strong inert sorbents (Tenax/Sulficarb), making them perfect for profiling a wide range of compounds over a wide volatility range, including reactive sulfur. Sampling was performed at a constant airflow of 100 mL min^−1^ for a period of 30 min.

### 2.2. Chemical Analyses

The analysis of VOCs was carried out as described in previous publications [2,9,10] using a thermal desorption-gas chromatography/mass spectrometry system (TD-GC/MS) consisting of a gas chromatograph/mass spectrometer (GC/MS-QP2020, Shimadzu, Japan) and a sulfur/labile thermal desorption unit (UNITY-xr, Markes International Limited, Llantrisant, UK). The analytical procedure is described in detail in the Supplementary Materials (Section S1).

### 2.3. Statistical Analysis

Statistical analyses were carried out using SPSS version 20.0 (IBM firm, Chicago, IL, USA). The non-parametric Mann-Whitney-U test was employed to examine statistically significant differences between samples. Spearman correlation coefficients were calculated to analyze correlations between various chemical groups of VOCs and meteorological conditions (ambient temperature, relative humidity, and wind speed). In all statistical analyses, *p* < 0.05 was considered statistically significant.

### 2.4. Quality Assurance/Control

All 117 targeted VOCs were detectable in the air samples; however, only 56 are reported here: those exhibiting frequencies of detection ≥ 50% and those where the percentage of values lower than the LOD were not exceeding 20%. Undetectable concentrations were considered equal to zero in the calculation of descriptive statistics. Concentrations lower than the LOD were assigned a value of LOD/2 for statistical analysis (Table S2). The studied compounds showed repeatabilities (% relative standard deviation values) ≤ 25%, achieving the EPA performance criteria [50]. Details about the reference standards and calibration curves method are provided in the Supplementary Materials (Section S2).

### 2.5. Risk Assessment

The inhalation non-cancer risk was evaluated by calculating the hazard quotient (HQ), i.e., (Equation (1)) the ratio of the exposure concentration in the air (EC mg m^−3^) (Equation (2)) to the inhalation reference concentration of individual VOCs (RfC, mg m^−3^) (Table 1). The total non-cancer health risk (THQ) was calculated by adding up the HQs of individual VOCs. If THQ > 1, the risk is unacceptable; if THQ < 1, the risk is acceptable [51].

The lifetime cancer risk (LCR) was calculated for benzene, which is classified as a “known” human carcinogen (Class A), for ethylbenzene, and for naphthalene. The inhalation cancer risk was calculated by multiplying the air concentrations by the compound’s Inhalation Unit Risk (IUR) using Equation (3). Toxicological parameters and values of variables used for the estimation of human health risk are given in Table 1 [52,53,54,55].

**Table 1:** Equations, non-cancer reference concentrations, cancer unit risks, toxicological parameters, and values of variables for the estimation of human health risk.

**Equations**				
HQ = EC/RfC				(1)
EC = (C × ET × EF × ED)/AT				(2)
LCR = EC × IUR				(3)
**Parameter**	**Physical Meaning**	**Units**	**Values**	
C	Concentration of VOC in air	μg m^−3^		
ET	Exposure time	hours day^−1^	8	
EF	Exposure frequency	days year^−1^	250	
ED	Exposure duration	y	50	
AT	Averaging time	years	25 years for general working period and 70 years for lifetime cancer risk assessment	
RfC	Inhalation reference concentration for chronic non-cancer health effects	mg m^−3^	0.03 for Benzene; 5 for Toluene; 1 for Ethylbenzene; 0.1 for m + p-Xylene; 1 for n-Propylbenzene; 0.06 for 1,3,5-Trimethylbenzene; 0.06 for 1,2,4-Trimethylbenzene; 0.06 for 1,2,3-Trimethylbenzene; 0.003 for Naphthalene; 1 for Pentane; 0.7 for Hexane; 0.4 for Heptane; 0.02 for Nonane; 0.008 for Propionaldehyde; 31 for Acetone; 0.03 for 4-Methyl-2-pentanone; 3 for 2-Hexanone [52,53,55]	
IUR	Inhalation unit risk	(μg m^−3^)^−1^	7.80 × 10^−6^ for Benzene; 2.50 × 10^−6^ for Ethylbenzene; 3.40 × 10^−5^ for Naphthalene [52,54]	

RfC: Non-cancer reference concentrations; IUR: Inhalation Unit Risk; [52] IRIS: USEPA Integrated Risk Information System; [53] ATSDR: Agency for Toxic Substances and Disease Registry; [54] Cal EPA: The California Environmental Protection Agency; [55] PPRTV: Provisional Peer-Reviewed Toxicity Values of IRIS.

## 3. Results

### 3.1. Concentrations of VOCs

The concentrations (μg m^−3^) of VOC compounds detected in indoor and outdoor air in Rhodes are provided in Table S3a, while summary statistics for concentrations (μg m^−3^) of the various chemical VOC classes are provided in Table S3b. The mean concentrations of ∑_56_VOCs in all sampling sites and for both sampling periods ranged between 30 and 1010 μg m^−3^ in the outdoor air and from 52 to 433 μg m^−3^ in the indoor air. The highest outdoor concentrations of ∑_56_VOCs were found in the Port site (P) (mean: 229 ± 121 μg m^−3^ in the cold period and 689 ± 288 μg m^−3^ in the warm period), probably due to the increased number of ships and cars, whereas the lowest were found in the urban background sampling site (UB) (mean: 46 ± 16 μg m^−3^ in the cold period and 127 ± 45 μg m^−3^ in the warm period). The highest indoor concentrations in the cold period were found in sampling site U1, while in the warm period at the Port (mean: 378 ± 53 μg m^−3^). The lowest mean concentrations in indoor air were found at sampling site U3 (mean: 140 ± 20 μg m^−3^ in the cold period and 155 ± 59 μg m^−3^ in the warm period).

The average outdoor concentrations of ∑_56_VOCs were higher in the warm period than in the cold period, although with statistically significant seasonal differences (*p* < 0.05) in sites U1, UB, P, and AIRP (Table S3). The highest cold/warm period ratios were observed for sampling site AIRP (4.6), followed by sampling sites P, UB, U1, and U2 (3.0, 2.6, 2.5, and 1.6, respectively). This may be due to the arrival of millions of tourists in the warm period, which causes a significant increase in the number of vehicles, planes, and ships. Also, high temperatures in summer lead to the increased release of VOCs from various sources. The average indoor concentrations of ∑_56_VOCs were also higher in the warm period compared to the cold period at all sampling sites, but a statistically significant difference was found only in sampling site P (*p* < 0.05), probably because the offices in the port of Rhodes were ventilated naturally and outdoor air entered indoors during cross-ventilation.

**Figure 2:**
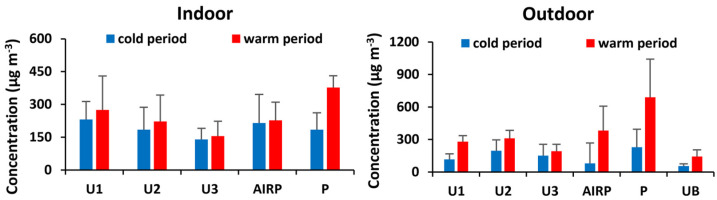
Sum concentrations of total VOC (∑_56_VOCs) in outdoor and indoor air in the cold and the warm period (mean ± SD) (n = 66).

The concentrations of total VOCs (∑_56_VOCs) in the cold and the warm periods for indoor and outdoor air are illustrated in Figure 2, while the summary statistics for concentrations of the various chemical classes of VOCs detected in indoor and outdoor air in Rhodes are presented in Table S3.

Results of the Spearman rank correlation analysis between outdoor VOC concentrations and meteorological parameters and indoor VOC levels and characteristics of indoor sampling sites and potential sources are shown in Table S4a,b, respectively. Significant positive correlations (*p* < 0.01) were observed in alkanes with ambient temperature. That may reflect the differences in the contribution of anthropogenic sources in different sampling periods. This would be consistent with short-chain alkane emissions from vehicles that were higher during the warm period than in the cold period. Other VOC classes, including aldehydes and ketones, exhibited significant negative correlations with relative humidity. No correlations were observed between the concentrations of VOCs and wind speed or direction, thus indicating possible local sources.

Factors associated with consumer products (electronics, furniture), materials, and other characteristics in the interior environment, such as ventilation, may affect indoor VOC levels. Correlation with the number of electronics and electrical equipment (EEE) and furniture was statistically significant, but only for aromatics and aldehydes (R^2^ = 0.68 and 0.74, respectively, *p* < 0.05) (Data from all sampling sites except the airport, because it was difficult to count all the furniture and electrical appliances in the airport). Finally, a statistically significant positive correlation (R^2^ = 0.69; 0.62 respectively, *p* < 0.05) was observed between acids and ketones and the type of ventilation (A/C or natural).

### 3.2. VOC Profiles

**Figure 3:**
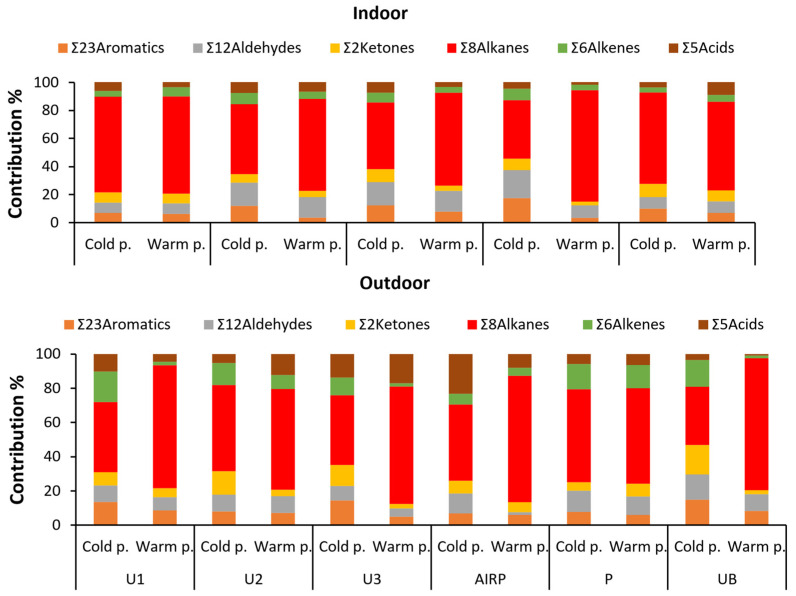
Mean profiles of VOC classes at the sampling sites of indoor and outdoor air during the cold and the warm sampling periods (23 aromatics, 12 aldehydes, 2 ketones, 8 alkanes, 6 alkenes, and 5 acids).

The profiles of VOC classes, i.e., the % contribution of each category to the ∑_56_VOCs in outdoor and indoor air, are provided in Figure 3.

In outdoor air, during the cold sampling period, ∑_8_Alkanes showed the highest contribution to the total VOCs (44%), followed by ∑_6_Alkenes (13%), ∑_12_Aldehydes, ∑_23_Aromatics, ∑_2_Ketones, and ∑_5_Acids (~11% for each one). A significant seasonal difference (*p* < 0.05) was observed for ∑_8_Alkanes, with an increased contribution (68%) in the summer. Alkanes are considered typical products of incomplete combustion processes of liquid fuels [18]. As mentioned above, during the warm period (tourist season), there is a significant increase in the number of cars (rental and non-rental), as well as ships (coasters, cruise ships, and ships from the neighboring coasts of Turkey) visiting the island of Rhodes. It should be noted that the large cruise ships that were in the port of Rhodes during the sampling period had their engines running to cover the energy needs of the tourists. The contribution of the remaining groups of VOCs in the warm period followed the order of ∑_5_Acids (8%) > ∑_12_Aldehydes (7%) > ∑_23_Aromatics (7%) > ∑_6_Alkenes (5%) > ∑_2_Ketones (5%).

For aldehydes and ketones, the dominant members were isobutyraldehyde, nonanal, decanal, and acetone in the cold sampling period (29%, 13%, 17%, and 95%, respectively) and in the warm sampling period (19%, 14%, 27%, and 95%, respectively). The profile of aromatics also shows a repeated pattern at the sampling sites, with BTEXs contributing ~90% of the total concentrations (Figure S1). For the alkanes, pentane and hexane were the compounds with the greatest contribution to the final concentration, and for alkenes, 1-pentene, 1-hexene, and 1-heptene (63% in the cold period and 91% in the warm period, respectively) were dominant. Finally, formic acid (57% in the cold period and 39% in the warm period) and acetic acid (25% in the cold period and 36% in the warm period) were the dominant acids.

In indoor air samples, hexane was the dominant congener for alkanes (62% and 76% for the cold and the warm period, respectively), and the compounds with the greatest contribution were 1-pentene, 1-hexene, 1-heptene, and 1-octene (75% in the cold period and 83% in the warm period, respectively). Finally, for aromatics, BTEXs contributed 83% (cold period) and 87% (warm period) of the total concentrations.

At most sampling sites, VOC concentration in indoor air was mainly dominated by three groups of VOCs: alkanes, aldehydes, and aromatic compounds, in both the cold and the warm sampling periods. Specifically, ∑_8_Alkanes showed the greatest contribution to total VOC concentrations, ranging from 42% to 80%, while the contribution of ∑_12_Aldehydes and ∑_23_Aromatics varied from 7% to 20% and from 3% to 17%, respectively. The remaining VOC groups followed the order of ∑_2_Ketones > ∑_6_Alkenes > ∑_5_Acids (Figure 3). No statistically significant differences were observed in the indoor VOC profiles between the cold and the warm sampling periods, suggesting that the emission sources of VOCs in the indoor environments were stable.

### 3.3. The Indoor/Outdoor Ratio

The indoor/outdoor ratio (I/O) is an indicator used to conclude whether pollutant concentrations in the indoor environment are due to indoor sources or to transportation from the outdoors. I/O ratio values ≪ 1 indicate that outdoor-air quality mainly determines indoor-air quality; I/O values ≈ 1 indicate that indoor and outdoor sources affect indoor air to the same extent; and finally, I/O values ≫ 1 indicate that indoor-emission sources are responsible for indoor-air concentrations [39]. Jia et al. [56] proposed the classification of VOCs in terms of I/O ratio into three groups: <1.5 ± 0.5, 1.5 to 10, or >10, indicating whether VOCs had primarily outdoor sources, both indoor and outdoor sources, or primarily indoor sources, respectively.

**Figure 4:**
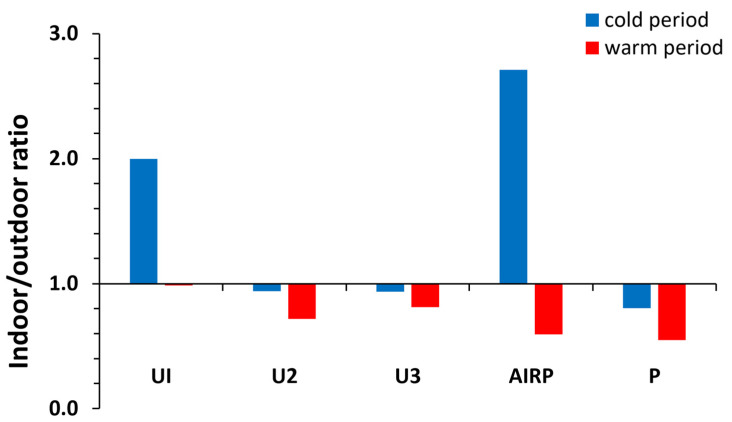
I/O ratios for total VOCs in the cold and the warm periods.

The I/O ratios from the sampling sites in Rhodes are represented in Figure 4 and Table 2. I/O ratios ranged from 0.80 to 2.76 and from 0.55 to 0.97 for ∑_56_VOCs in the cold and the warm periods, respectively. About 80% of the I/O ratios of ∑_56_VOCs in the cold period were ≥1, both indoor and outdoor sources, whereas 100% of I/O ratios in the warm period were less than 1, which means that the emissions of VOC compounds were mainly due to outdoor sources. More specifically, for sampling sites U1, U2, and U3, in the cold period, I/O values indicate that VOCs had both indoor and outdoor sources for most VOC groups, while I/O ratios were less than 1 in the warm period, except for alkenes and aldehydes in the U1 and U3 sampling sites. Aldehydes and alkenes in indoor air can be identified as human-body emissions [57]. In the AIRP sampling site, I/O ratios were >1 for all VOC groups in the cold period, except acids, while in the warm period, I/O ratios were <1 except for aldehydes. In the cold season, activity at the airport is minimal, and the main sources of VOCs are indoors (solvents, cleaning products, consumer products, etc.), while in the warm season, when the operation of the airport is intense, the main sources are outdoors, especially the use of fuels for the airplanes, support vehicles, and the fleet of rental cars, etc. [48]. In the port, I/O ratios were less or close to 1 for both sampling periods, indicating predominantly outdoor sources, such as the movement of ships, vehicles, and cargo vehicles [46,47]. De Blas et al. [58] reported that BTEX and alkanes (hexane), well known as traffic-related VOCs, had an I/O ratio < 1. Also, similar I/O ratios were reported at office buildings in Kuwait for aromatics and carbonyl compounds [59].

In the warm period, the I/O ratios were lower than in the cold period for the majority of VOC compounds. Specifically, in the cold period, 20 compounds (36% of the total compounds) had an I/O ratio < 1.5, and 34 compounds (61%) had a ratio of between 1.5 and 10, which indicated indoor and outdoor sources; in the warm period, 37 compounds (66%) had an I/O ratio < 1.5, and 19 compounds (34%) had an I/O ratio of between 1.5 and 10. In the cold period, higher I/O values were observed in larger alkanes (heptane, octane, nonane, decane, undecane, dodecane) (I/O ratio 5.8–13.6). VOCs with I/O values < 1.5 were alkanes (pentane, hexane), alkenes (1-pentene, I hexane, 1-heptane, 1-octane), aromatics (benzene, toluene, pentamethylbenzene, 2-methylnaphthalene, 1-methylnaphthalene), carbonyl compounds (butyraldehyde, isovaleraldehyde, decanal, acetone), and acids (formic acid, acetic acid). The rest of the aromatics showed an I/O ratio between 1.5 and 10. In the warm period, the outdoor sources were dominant (I/O ratio < 1.5 for the majority of compounds). Only C7–C12 alkanes, 1-hexene, 1-heptane, 1-decene, isopropylbenzene, n-propylbenzene, n-heptanal, nonanal, undecanal, dodecanal, hexanoic acid, and acetone had I/O ratios between 1.5 and 10.

### 3.4. Source Identification

The Positive Matrix Factorization (PMF) model is a receptor model that shows the source profile and source contribution of pollutants [60]. PMF has been extensively used to identify sources of VOCs since the PMF model does not require the input of source profiles [61]. In this study, the PMF model (v.5) was used to quantify the contribution of potential emission sources of VOCs [62,63]. Two input files are required by PMF: sample-species concentration values and sample-species uncertainty values, or parameters for calculating uncertainty. The estimation method of uncertainties for VOC data originated from US EPA PMF guidelines. The PMF model is described in detail in the Supplementary Materials (Section S3).

The marker species for VOC sources were extremely complex, and the same species can be used to identify different source categories (such as benzene, toluene, etc.). Also, VOC compounds with high reactivity can result in inaccurate outcomes due to varying photochemical reaction losses [64]. The determination of the number of factors has a major impact on the source-analysis results. The selection of the number of factors in this study was based on a correlation between the observed and calculated TVOC or species concentrations and Q_true_/Q_theo_ ratios [65]. Bootstrap was performed, and 100 runs were performed using random seeds with a minimum Pearson correlation coefficient of 0.6. All modelling factors were well mapped in at least 90% of the operation. We acknowledge the limitation of this study—that the small sample size may lead to uncertainties in the PMF model—but the results were still helpful for understanding outdoor and indoor VOC sources. 

For outdoor and indoor samples, four factors, respectively, were finally selected for this study. These factors were attributed to sources based on dominant species and correlations among several compounds. Source profiles from the base run and bootstrapping analysis are shown in Figure S2 for outdoors and in Figure S3 for indoors.

Outdoors

Factor 1: Solvent use

Factor 1 was rich in aromatic compounds (trimethylbenzene and TEX (toluene, ethylbenzene, m/p-xylene and o-xylene)). TEX is often used as a solvent in paints, coatings, synthetic fragrances, adhesives, inks, and cleaning agents, in addition to its use in fossil fuels [66,67,68,69]. Also, 1,2,4-trimethylbenzene, 1,3,5-trimethylbenzene, and acetone are typical tracers of solvent usage [66,67,68,69,70,71,72].

Factor 2: Traffic emissions

Factor 2 had contributions to almost all measured VOC species. It was rich in aromatic compounds (benzene; toluene; m + p-xylene; o-xylene; ethylbenzene; 1,2,4-trimethylbenzene; 1,3,5-trimethylbenzene; 1-methyl-3-ethylbenzene), aldehydes (nonanal; decanal; undecanal; dodecanal), alkanes (pentane; hexane), and alkenes (1-pentene; 1-hexene). Factor 2 was identified as vehicle emissions. BTEX are the key tracers of gasoline combustion in mobile motors. BTEX and n-hexane sources may be fossil fuel combustion from traffic-related or industrial sources, while they are good tracers for diesel vehicle emissions [73,74,75,76]. Also, higher alkanes (nonane, decane, undecane, dodecane), trimethylbenzene isomers (1,3,5-trimethylbenzene, 1,2,4-trimethylbenzene), xylene isomers, ethylbenzene, propylbenzene, and butylbenzene are typically emitted from diesel vehicles [77,78,79]. Finally, propionaldehyde was detected in commercial jet aircraft exhaust [80].

Factor 3: Industrial and vehicular evaporative emissions

Factor 3 was interpreted to be industrial and vehicular evaporative emissions characterized by high levels of alkene species [81], pentane, hexane, heptane, octane, and acetone [70,82]. 

Aromatic species (i.e., toluene, o-xylene, m+ p-xylenes) and larger alkanes were consistent with tailpipe or gasoline-evaporative emissions [83,84,85], while C6–C7 alkanes were likely the result of diesel-fuel evaporation [78,86].

Factor 4: Aged emissions

Factor 4 mainly consists of longer-lived and less-reactive VOC species among the measured VOCs, such as alkanes and benzene [87,88]. Moreover, the minimal contribution of reactive VOC compounds, such as alkenes, in Factor 4 indicates that VOC sources may be at long distances from the sampling sites. Factor 4 also contains carbonyl compounds. The presence of aldehydes in factor 4 indicates their formation in the atmosphere as a result of photochemical oxidation of reactive hydrocarbons [89].

Indoors

Factor 1: Household products and solvent-related emissions

Factor 1 was distinguished by aromatics, alkenes, and acetone. In general, those compounds are a major constituent of solvents and consumer products [26,31,74,90,91]. BTEX, trimethylbenzenes, octane, and nonane are common components of water-based latex paints and solvent-based paints [70]. Acetone also can be used in personal-care products [23].

Factor 2: Human VOC emissions from skin and breath

Factor 2 was distinguished by octanal, nonanal, decanal, undecanal, dodecanal, acetone, and alkenes. Those compounds were identified as human body emissions from bodily fluids, skin, and as human breath emissions [57,92,93,94,95].

Factor 3: Outdoor sources

Factor 3 consisted of alkanes (pentane, hexane, octane) and aromatics (BTEX, 1,2,4-trimethylbenzene, 1,2,3-trimethylbenzene). Aromatic compounds and alkanes were a major constituent in solvents, vehicle exhausts, and industrial sources. The higher contribution rates in factor 3 than in other factors could be mostly attributed to outdoor sources [90,96].

Factor 4: Wood-based product emissions

Factor 4 was rich in carbonyl compounds. Common indoor sources for carbonyl compounds are wood-based furniture and materials, wall paints, wall and floor coverings such as carpets (with latex backing), and personal-care cleaning products [97,98,99,100,101]. Also, nonane, n-decane, undecane, dodecane, and ethylbenzene are common in indoor sources such as petroleum-based indoor coating, wood stain, polyurethane wood finish, and floor wax [102].

**Figure 5:**
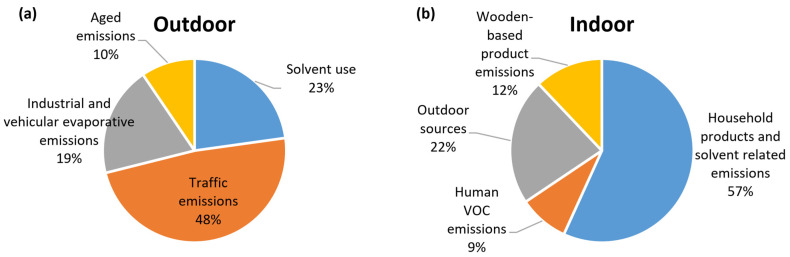
Average source contribution to total measured VOCs for outdoor (**a**) and indoor (**b**) environments.

Figure 5a,b illustrates the mass contribution of each source to the sum of species for outdoor and indoor samples, respectively. For outdoor environment traffic emissions (48%), solvent use (23%) and industrial and vehicular evaporative emissions (19%) were the dominant outdoor sources of VOCs. For indoor environments, 57% of indoor VOC concentrations were attributed to household products and solvent-related emissions. The remainder was apportioned to outdoor sources (ventilation/infiltration; 22%), wood-based product emissions (12%), and human VOC emissions from skin and breath (9%). It can be concluded that indoor human-related emissions (human activities and human body emissions) were responsible for a huge percentage of the indoor VOC concentrations.

### 3.5. Health Risk

The assessment of potential non-carcinogenic risk was performed only for 9 VOC members (benzene; toluene; ethylbenzene; m + p-xylene; n-propylbenzene; 1,3,5-trimethylbenzene; 1,2,4-trimethylbenzene; 1,2,3-trimethylbenzene; naphthalene; pentane; hexane; heptane; nonane; propionaldehyde; acetone; 4-methyl-2-pentanone; and 2-hexanone) for which RfC values are available in the literature (Table 3a).

Non-cancer risk for adults from exposure to VOCs in outdoor air followed the order of hexane > nonane > naphthalene > propionaldehyde > benzene> m + p-xylene > heptane. The corresponding order in indoor air was nonane> hexane> naphthalene> heptane> propionaldehyde > m + p-xylene > benzene. The THQ in all cases was less than 1, indicating that exposure to VOCs in outdoor and indoor air does not pose a potential health risk to adults.

The LCR risk was calculated for three aromatic hydrocarbons: benzene, which is classified as a ‘known’ human carcinogen (class A), ethylbenzene, and naphthalene. For these compounds, IUR risk unit values were available in the literature. Median LCR values for adults followed the order of naphthalene > benzene > ethylbenzene in both outdoor and indoor air. According to previous studies [103,104,105], the following classification for the carcinogenic risk assessment was applied in the present study: *R* ≤ 1 × 10^−6^ suggests that the carcinogenic risk can be ignored; 1 × 10^−6^ < *R* ≤ 1 × 10^−4^ suggests a minor carcinogenic risk; 1 × 10^−4^ < *R* < 1 × 10^−3^ suggests a moderate carcinogenic risk; and *R* ≥ 1 × 10^−3^ suggests a significant carcinogenic risk. The overall carcinogenic risk for the 3 VOCs in the present study was found to be within the safe range (Table 3b).

## 4. Discussion

The purpose of this study was to measure and evaluate the levels of VOCs on Rhodes Island in both outdoor and indoor air. The highest concentrations in the outdoor and indoor air were found in the port of Rhodes. The increased concentrations of total VOCs in the warm period compared to the cold period are probably due to the increase in cars and ships that visit Rhodes during the tourist season. ∑_8_Alkanes showed the greatest contribution to total VOC concentrations in outdoor and indoor samples. The findings of this study indicate that the levels of VOCs in outdoor air on Rhodes Island are considerably affected by location and by sampling period. In the warm period, the high outdoor VOC content very strongly affected the indoor air. The PMF model was applied to better understand and characterize different VOC sources in outdoor and indoor environments. Traffic emissions were the dominant outdoor sources of VOCs, while human-related sources were dominant indoors. In all cases, the concentrations of VOC compounds were lower than the RfC, while the overall carcinogenic risk for the three studied compounds (benzene, ethylbenzene, and naphthalene) was found to be within the safe range. Overall, although the findings of the present study improve our understanding of VOC behavior and sources, additional research is needed. Since VOCs have been found responsible for various health problems in humans, large-scale studies are required to comprehend indoor and outdoor VOC pollution in urban sites and the impact of long-term exposure on humans. The results may assist policymakers in improving indoor- and outdoor-air-quality management.

## Figures and Tables

**Table 2:** I/O ratios for VOCs groups (I/O ratio > 1 are shown in bold italics).

VOCs Group	Sampling Period	UI	U2	U3	AIRP	P
∑_23_Aromatics	cold period	***1.0***	***1.4***	0.8	***6.9***	***1.1***
∑_12_Aldehydes		***1.5***	***1.6***	***1.8***	***4.6***	0.5
∑_2_Ketones		***1.8***	0.4	0.7	***3.0***	***1.5***
∑_8_Alkanes		***3.3***	0.9	***1.1***	***2.5***	1.0
∑_6_Alkenes		0.5	0.6	0.6	***3.6***	0.2
∑_5_Acids		***1.2***	***1.3***	0.5	0.5	0.5
∑_56_VOCs		***2.0***	1.0	1.0	***2.8***	0.8
∑_23_Aromatics	warm period	0.7	0.4	***1.3***	0.3	0.6
∑_12_Aldehydes		1.0	***1.1***	***2.5***	***3.2***	0.4
∑_2_Ketones		***1.3***	0.9	***1.1***	0.3	0.6
∑_8_Alkanes		0.9	0.8	0.8	0.6	0.6
∑_6_Alkenes		***3.0***	0.5	***1.8***	0.5	0.2
∑_5_Acids		0.8	0.4	0.2	0.1	0.8
∑_56_VOCs		0.9	0.7	0.8	0.6	0.6

**Table 3:** (**a**). Non-cancer risk for adults due to inhalation of VOCs. (**b**). Cancer risk for adults due to inhalation of VOCs.

**(a)**	**Non-Cancer Risk HQ**					
	**Indoor Workers**			**Outdoor Workers**		
**VOCs**	**5th Percentile**	**Median**	**95th Percentile**	**5th Percentile**	**Median**	**95th Percentile**
Benzene	3.22 × 10^−5^	2.06 × 10^−4^	8.20 × 10^−4^	4.86 × 10^−5^	1.95 × 10^−4^	5.12 × 10^−4^
Toluene	6.13 × 10^−6^	6.01 × 10^−5^	2.52 × 10^−4^	9.34 × 10^−6^	7.15 × 10^−5^	3.82 × 10^−4^
Ethylbenzene	9.95 × 10^−7^	1.23 × 10^−5^	9.91 × 10^−5^	9.95 × 10^−7^	5.66 × 10^−6^	5.59 × 10^−5^
m + p-Xylene	2.75 × 10^−5^	2.95 × 10^−4^	3.06 × 10^−3^	3.68 × 10^−5^	1.61 × 10^−4^	1.58 × 10^−3^
n-Propylbenzene	7.49 × 10^−7^	3.90 × 10^−6^	2.58 × 10^−5^	4.16 × 10^−7^	2.05 × 10^−6^	1.14 × 10^−5^
1,3,5-Trimethylbenzene	9.72 × 10^−6^	5.83 × 10^−5^	3.94 × 10^−4^	9.13 × 10^−6^	3.38 × 10^−5^	1.84 × 10^−4^
1,2,4-Trimethylbenzene	2.92 × 10^−5^	1.70 × 10^−4^	1.04 × 10^−3^	1.69 × 10^−5^	8.24 × 10^−5^	4.72 × 10^−4^
1,2,3-Trimethylbenzene	1.18 × 10^−5^	9.22 × 10^−5^	3.24 × 10^−4^	6.09 × 10^−6^	3.52 × 10^−5^	2.22 × 10^−4^
Naphthalene	3.63 × 10^−4^	1.37 × 10^−3^	2.08 × 10^−2^	1.38 × 10^−4^	5.66 × 10^−4^	5.17 × 10^−3^
Pentane	1.79 × 10^−5^	8.21 × 10^−5^	5.42 × 10^−4^	1.04 × 10^−5^	5.33 × 10^−5^	7.19 × 10^−4^
Hexane	5.20 × 10^−4^	5.87 × 10^−3^	1.44 × 10^−2^	3.97 × 10^−4^	6.20 × 10^−3^	1.65 × 10^−2^
Heptane	5.96 × 10^−6^	4.28 × 10^−4^	2.76 × 10^−3^	1.08 × 10^−5^	1.25 × 10^−4^	2.32 × 10^−3^
Nonane	1.28 × 10^−3^	9.71 × 10^−3^	4.70 × 10^−2^	4.21 × 10^−4^	3.61 × 10^−3^	5.46 × 10^−2^
Propionaldehyde	-	3.22 × 10^−4^	1.11 × 10^−2^	-	3.46 × 10^−4^	2.63 × 10^−2^
Acetone	2.65 × 10^−6^	1.10 × 10^−5^	8.64 × 10^−5^	7.96 × 10^−7^	7.63 × 10^−6^	9.03 × 10^−5^
2-Hexanone	0	3.63 × 10^−6^	4.99 × 10^−5^	2.84 × 10^−7^	2.77 × 10^−6^	2.49 × 10^−5^
THQ	2.31 × 10^−3^	1.87 × 10^−2^	1.03 × 10^−1^	1.11 × 10^−3^	1.15 × 10^−2^	1.09 × 10^−1^
**(b)**	**Cancer Risk RC**					
	**Indoor Workers**			**Outdoor Workers**		
**VOCs**	**5th Percentile**	**Median**	**95th Percentile**	**5th Percentile**	**Median**	**95th Percentile**
Benzene	2.69 × 10^−8^	1.72 × 10^−7^	6.86 × 10^−7^	4.06 × 10^−8^	1.63 × 10^−7^	4.28 × 10^−7^
Ethylbenzene	8.88 × 10^−9^	1.10 × 10^−7^	8.85 × 10^−7^	8.88 × 10^−9^	5.05 × 10^−8^	4.99 × 10^−7^
Naphthalene	1.32 × 10^−7^	5.00 × 10^−7^	7.58 × 10^−6^	5.03 × 10^−8^	2.06 × 10^−7^	1.88 × 10^−6^
Total cancer risk	1.68 × 10^−7^	7.82 × 10^−7^	9.15 × 10^−6^	9.98 × 10^−8^	4.20 × 10^−7^	2.81 × 10^−6^

## Data Availability

The original contributions presented in the study are included in the article/Supplementary Materials, further inquiries can be directed to the corresponding author/s.

## Supplementary Materials

The following supporting information can be downloaded at https://www.mdpi.com/article/10.3390/toxics12070486/s1, Section S1: TD/GC-MS analysis; Section S2: Quality assurance and control; Section S3: PMF model (v.5); Table S1a: Summary of sampling information; Table S1b: Characteristics of indoor sampling sites and potential sources of VOCs; Table S2: LODs and LOQs of VOCs detected in indoor and outdoor air with frequencies ≥ 50%; Table S3a: Concentrations (μg m^−3^) of VOC compounds detected in indoor and outdoor air in Rhodes; Table S3b: Summary statistics for concentrations (μg m^−3^) of the various chemical classes of VOCs detected in indoor and outdoor air in Rhodes; Table S4a: Spearman’s rho correlation coefficients between the chemical classes of outdoor VOCs and meteorological data; Table S4b: Spearman correlation coefficients between the chemical classes of indoor VOCs and characteristics of indoor sampling sites and potential sources; Figure S1: Profiles of VOC categories in indoor and outdoor air; Figure S2: Outdoor PMF-resolved VOC source profiles (% of species); Figure S3: Indoor PMF-resolved VOC source profiles (% of species). References [2,9,10] are cited in the Supplementary Materials.
