# Supplementary material for: Volatile Organic Compounds on Rhodes Island, Greece: Implications for Outdoor and Indoor Human Exposure
Source: Toxics. 2024 Jul 2;12(7):486. doi: 10.3390/toxics12070486 (PMC11280855; doi:10.3390/toxics12070486)
Supplement: Supplementary file 1 [file toxics-12-00486-s001.zip › toxics-3029659 - supplementary materials.pdf]

Volatile Organic Compounds in Rhodes Island, Greece. Implications for outdoor and indoor human exposure

Athanasios Besis [1,2,*], Dimitrios Margaritis [1], Constantini Samara [2] and Evangelos Bekiaris [1]

[1] Centre for Research and Technology Hellas (CERTH) / Hellenic Institute of Transport (HIT)

[2] Environmental Pollution Control Laboratory, Department of Chemistry, Aristotle University of Thessaloniki, GR-54124, Thessaloniki, Greece

* Correspondence: Athanasios Besis; athanasb@chem.auth.gr; Tel.:+30-231-099-7858

Section S1: TD/GC-MS analysis

TD/GC-MS analysis

Briefly, the primary thermal desorption of VOCs was performed at 200 °C at a He (99.999% or higher purity) flow rate of 50 mL min^{-1} for 3 min. The cold trap (U-T6SUL-26, Markes International Limited, Llantrisant, UK) connected in line was maintained at -10 °C. After primary desorption, the cold trap was rapidly heated from -10 °C to 200 °C and maintained at this temperature for 3 min (secondary desorption). Analytes were then injected into the capillary column (MEGA-624 MS PLUS, 60 m × 0.25 mm × 1.4 μm) for chromatographic separation via the transfer line at 200 °C. The column oven temperature started at 40 °C for 1 min, increased to 230 °C at a rate of 6 °C min^{-1}, and then was maintained at 230 °C for 15.10 min. Helium (99.999%) carrier gas flow the analytical column was approximately 1.5 mL min^{-1} (total program time 47.8 min). Electron impact source was obtained with electron energy of 70 eV. Mass spectral data were acquired over a mass range of 42-300 amu. Ion source and interface temperature were set at 230 °C and 240 °C, respectively. The TD-GC/MS system was calibrated using liquid custom standards (mixtures and individual compounds in MeOH, toluene, or isooctane) [1–3].

The Markes' Calibration Solution Loading Rig (CSLR), specifically designed for loading sorbent tubes with liquid-phase standards, was used in the TD-GC/MS system. Standard solution was prepared so that the mass of analytes introduced in the injection volume matches the masses collected during in-cabin sampling. The liquid calibration standard (1.0 μL) was injected through the injector septum using a precision syringe, and onto the sampling end of the attached tube. The solution vaporized in the carrier gas flow (nitrogen, 50 mL min^{-1} for 3-5 min) and was passed onto the sorbent tube in the vapor phase. A sufficient volume of carrier gas was required to pass through the tube so that most of the carrier solvent (e.g., methanol) passes through the sorbent and away to vent while the compounds of interest are still quantitatively retained. Then, the tube was placed directly in the tube oven for analysis [1–3].

The qualitative identification of VOCs was based on retention time and the match of the ion ratios of the target qualifier ions, using the MS data of the NIST-14 mass spectra library (NIST/EPA/NIH, NIST MS Search version 14d). A chromatographic peak was assigned to a specific VOC when the retention time

was the same as that of the standard compound by ±2 s. The mass spectrum obtained from the various members of a class of isomers was particularly characteristic, thus facilitating the identification of the various members. The GC/MS variables were optimized for the simultaneous determination of 117 VOCs including alkanes, alkenes, aromatics, aldehydes, ketones, organic acids, sulfides, mercaptans, thiophenes, phenols and amines.

Section S2: Quality assurance and control

The Markes' Calibration Solution Loading Rig (CSLR) that has been specifically designed for loading sorbent tubes with liquid-phase standards, was used. Standard solution was prepared so that the mass of analytes introduced in the injection volume matches the expected masses that will be collected during field monitoring. The liquid calibration standard (1.0 μL) was injected through the injector septum using a precision syringe, and onto the sampling end of the attached tube. The solution vaporized in the carrier gas flow (nitrogen, 50 mL min^{-1} for 3-5 min) and was passed onto the sorbent tube in the vapor phase. A sufficient volume of carrier gas was required to pass through the tube so that most of the carrier solvent (e.g., methanol) passes through the sorbent and away to vent while the compounds of interest are still quantitatively retained. Then, the tube was placed directly in the tube oven for analysis [1–3].

The qualitative identification of VOCs was based on retention time and the match of the ion ratios of the target qualifier ions, using the MS data of the NIST-14 mass spectra library (NIST/EPA/NIH, NIST MS Search version 14d). A chromatographic peak was assigned to a specific VOC when the retention time was the same as that of the standard compound by ±2s. The mass spectrum obtained from the various members of a class of isomers was particularly characteristic, thus facilitating the identification of the various members.

Quantification of VOCs was conducted by the external standard method using 4-point calibration curves (excluding the blank) that were constructed for each one of the 56 identified compounds. The standard concentrations covered, at least, the range of concentrations encountered during the analysis of real samples. Calibration curves were tested on a daily basis. Limits of detection (LOD) were calculated as signal-to- noise ratio of 3 and the limits of quantification (LOQ) as three times the LOD.

Section S3: PMF model (v.5)

Receptor models are mathematical approaches for quantifying the contribution of sources to samples based on the composition or fingerprints of the sources. The composition or speciation is determined using analytical methods appropriate for the media, and key species or combinations of species are needed to separate impacts. A speciated data set can be viewed as a data matrix X of i by j dimensions, in which i number of samples and j chemical species were measured, with uncertainties u. The goal of receptor models is to solve the chemical mass balance (CMB) between measured species concentrations and source profiles, as shown in Equation S1, with number of factors p, the species profile f of each source, and the amount of mass g contributed by each factor to each individual sample (Equation S1):

$$x_{ij} = \sum_{k=1}^{p} g_{ik} f_{kj} + e_{ij} \quad \text{(S1)}$$

where *eij* is the residual for each sample/species. The CMB equation can be solved using multiple models including EPA CMB, EPA Unmix, and EPA Positive Matrix Factorization (PMF).

PMF is a multivariate factor analysis tool that decomposes a matrix of speciated sample data into two matrices: factor contributions (G) and factor profiles (F). These factor profiles need to be interpreted by the user to identify the source types that may be contributing to the sample using measured source profile information, and emissions or discharge inventories.

Factor contributions and profiles are derived by the PMF model minimizing the objective function Q as follows:

$$Q = \sum_{i=l}^{n} \sum_{j=l}^{m} \left[\frac{x_{ij} - \sum_{k=1}^{p} g_{ik} f_{kj}}{u_{ij}} \right] \quad \text{(S2)}$$

Q is a critical parameter for PMF and two versions of Q are displayed for the model runs.

Q(true) is the goodness-of-fit parameter calculated including all points.

Q(robust) is the goodness-of-fit parameter calculated excluding points not fit by the model, defined as samples for which the uncertainty-scaled residual is greater than 4.

Data

The missing concentration data were replaced by the geometric mean of the detected concentration while the uncertainty file was prepared following the recommendation from U.S EPA (U.S. EPA, 2014). For model input, the uncertainty data was calculated based on USEPA FMF user guide (Eq. S3). The concentration sample data uncertainty is calculated using the following equation.

Additionally, where more than 25% of missing data occurred, or data values were lower than the method detection limit, (MDL), these compounds were rejected from the PMF analysis in this study.

$$Unc = \sqrt{(Error\ Fraction \times concentration)^2 + (0.5 \times MDL)^2} \quad (conc > MDL) \quad \text{(S3)}$$

When the concentrations were below or equal to MDL the Eq. S4 was used.

$$Unc = \frac{5}{6} \times MDL \quad (conc \leq MDL) \quad \text{(S4)}$$

where, MDL represents the method detection limit, error fraction. The error fraction in this study was 10%.

Bootstrap was performed, and 100 runs are performed, using random seeds, with a minimum Pearson correlation coefficient of 0.6. All modelling factors were well mapped in at least 90% of the operation.

Table S1a. Summary of **meteorological data during** sampling

	A/A	sampling date	Temperature (^{O}C)	RH (%)	Wind speed ($m\ s^{-1}$)	Wind direction
**Cold period**	1	21/3/2023	17.7	71	3.3	SW
	2	22/3/2023	17.8	67	2.7	E
	3	23/3/2023	19.5	60	3.2	E
**Warm period**	5	25/7/2023	33.2	33	2.3	NE
	6	26/7/2023	33.9	34	2.0	NE
	7	27/7/2023	31.0	31	2.5	NE

Table S1b. Characteristics of indoor sampling sites and potential sources of VOCs

A/A	Sample code	Sampling site	Area (m^2)	Height (m)	No. of furniture and EEE[a]	No. of fireplaces	No. of smokers	Cooking	Ventilation
1	U1	Museum	180	4.5	6	0	0	N	AC
2	U2	Hotel	22	3	15	0	0	N	AC
3	U3	Offices	15	3	11	0	0	N	AC
4	P	Port	75	3	23	0	0	N	natural
5	AIRP	Airport	49.478	12	>500*	0	0	Y	AC

[a]EEE: Electronic & electric equipment ; [b]AC: air conditioner; N: NO; Y: YES

***We assume >500 because it was difficult to count all the furniture and electrical appliances in the airport.**

Table S2. LODs, LOQs and MDLs of VOCs detected in indoor and outdoor air with frequencies ≥ 50%

VOCs	CAS No.	LOD (ng μL^{-1})	LOQ (ng μL^{-1})	MDL (μg m^{-3})
**Alkanes**				
Pentane	109-66-0	0.0707	0.2333	0.0236
Hexane	110-54-3	0.4002	1.3207	0.1334
Heptane	142-82-5	0.008	0.0264	0.0027
Octane	111-65-9	0.0047	0.0155	0.0016
Nonane	111-84-2	0.0062	0.0205	0.0021
Decane	124-18-5	0.0076	0.0251	0.0025
Undecane	1120-21-4	0.0291	0.096	0.0097
Dodecane	112-40-3	0.0063	0.0208	0.0021
**Alkenes**				
1-Pentene	109-67-1	0.2448	0.8078	0.0816
1-Hexene	592-41-6	0.2135	0.7046	0.0712
1-Heptene	592-76-7	0.0371	0.1224	0.0124
1-Octene	111-66-0	0.1109	0.366	0.0370
1-Nonene	124-11-8	0.0124	0.0409	0.0041
1-Decene	872-05-9	0.0293	0.0967	0.0098
**Aromatics**				
Benzene	71-43-2	0.0003	0.001	0.0001
Toluene	108-88-3	0.0014	0.0046	0.0005
Ethylbenzene	100-41-4	0.008	0.0264	0.0027
m+p-Xylene	108-38-3	0.0084	0.0277	0.0028
o-Xylene	95-47-6	0.0134	0.0442	0.0045
Isopropylbenzene	98-82-8	0.0007	0.0023	0.0002
n-Propylbenzene	103-65-1	0.0028	0.0092	0.0009
1-Methyl-3-ethylbenzene	620-14-4	0.0007	0.0023	0.0002
1-Methyl-4-ethylbenzene	622-96-8	0.0009	0.003	0.0003
1,3,5-Trimethylbenzene	108-67-8	0.01	0.033	0.0033
1-Methyl-2-ethylbenzene	611-14-3	0.0091	0.03	0.0030
1,2,4-Trimethylbenzene	95-63-6	0.0115	0.038	0.0038
1,2,3-Trimethylbenzene	526-73-8	0.0059	0.0195	0.0020
Indan	496-11-7	0.0028	0.0092	0.0009
1,4-Diethylbenzene	105-05-5	0.0047	0.0155	0.0016
n-Butylbenzene	104-51-8	0.0058	0.0191	0.0019

VOCs	CAS No.	LOD (ng μL^{-1})	LOQ (ng μL^{-1})	MDL (μg m^{-3})
1,2-Diethylbenzene	135-01-3	0.0065	0.0215	0.0022
1,2,4,5-Tetramethylbenzene	95-93-2	0.0018	0.0059	0.0006
1,2,3,5-Tetramethylbenzene	527-53-7	0.0057	0.0188	0.0019
Naphthalene	91-20-3	0.0008	0.0026	0.0003
Pentamethylbenzene	700-12-9	0.0024	0.0079	0.0008
2-Methylnaphthalene	91-57-6	0.1229	0.4056	0.0410
1-Methylnaphthalene	90-12-0	0.013	0.0429	0.0043
**Aldehydes**				
Propionaldehyde	123-38-6	0.0038	0.0125	0.0013
Isobutyraldehyde	50-84-2	0.0028	0.0092	0.0009
Butyraldehyde	123-72-8	0.0275	0.0908	0.0092
Isovaleraldehyde	590-86-3	0.0046	0.0152	0.0015
n-Hexanal	66-25-1	0.0544	0.1795	0.0181
n-Heptanal	111-71-7	0.0098	0.0323	0.0033
Benzaldehyde	100-52-7	0.0031	0.0102	0.0010
Octanal	124-13-0	0.0887	0.2927	0.0296
Nonanal	124-19-6	0.1188	0.392	0.0396
Decanal	112-31-2	0.1033	0.3409	0.0344
Undecanal	112-44-7	0.1229	0.4056	0.0410
Dodecanal	112-54-9	0.0885	0.2921	0.0295
**Ketones**				
Acetone	67-64-1	0.8275	2.7308	0.2758
2-Hexanone	108-10-1	0.0118	0.0389	0.0039
**Acids**				
Formic acid	64-18-6	0.0594	0.196	0.0198
Acetic acid	64-19-7	0.0617	0.2036	0.0206
Propionic acid	79-09-4	0.0108	0.0356	0.0036
Isobutyric acid	79-31-2	0.0005	0.0017	0.0002
Hexanoic acid	142-62-1	0.6073	2.0041	0.2024

Table S3a: Concentrations (μg m^{-3}) of VOC compounds detected in indoor and outdoor air in Rhodes

	INDOOR SAMPLES														
	COLD PERIOD														
	U1			U2			U3			P			AIRP		
VOCs	21/3/2023	22/3/2023	23/3/2023	21/3/2023	22/3/2023	23/3/2023	21/3/2023	22/3/2023	23/3/2023	21/3/2023	22/3/2023	23/3/2023	21/3/2023	22/3/2023	23/3/2023
Pentane	3.46	6.09	2.98	0.78	0.89	1.87	2.15	1.56	1.87	0.94	1.18	1.66	0.73	4.34	3.26
Hexane	173.09	101.77	62.16	22.52	89.07	67.48	31.07	49.60	60.13	101.53	113.87	46.77	12.28	48.09	40.86
Heptane	18.71	5.54	3.97	1.81	4.60	4.60	1.17	7.60	2.85	6.53	4.94	4.10	0.13	6.17	3.62
Octane	6.82	7.13	8.68	8.17	5.69	10.28	2.89	3.05	3.49	11.89	6.87	2.93	0.25	10.53	6.21
Nonane	12.57	10.09	7.66	1.60	4.31	7.49	3.10	2.31	3.22	5.01	10.63	7.52	2.01	13.10	9.63
Decane	4.49	10.06	5.04	2.20	4.89	4.44	3.16	2.13	3.18	4.93	5.02	7.44	1.68	23.50	15.11
Undecane	5.66	5.91	0.83	2.90	8.12	5.54	5.80	1.77	2.16	2.68	3.37	2.00	0.88	25.10	15.11
Dodecane	3.46	3.34	3.19	2.03	9.75	4.96	2.85	1.25	1.76	2.43	3.43	2.47	0.67	16.12	9.82
$\sum_{8}$Alkanes	228.27	149.93	94.51	42.00	127.32	106.65	52.80	68.77	78.55	135.94	149.29	74.88	18.94	146.51	103.71
1-Pentene	1.26	0.86	0.78	0.43	0.45	3.10	5.04	1.48	0.47	0.58	1.40	2.59	0.77	1.10	1.48
1-Hexene	1.38	12.81	0.12	2.00	2.71	2.39	2.44	1.80	2.83	1.75	3.84	1.99	0.32	1.01	0.93
1-Heptene	1.33	1.69	1.33	0.02	0.01	0.01	0.92	1.05	1.12	0.00	0.23	0.00	0.03	0.00	0.04
1-Octene	0.57	1.36	0.04	0.28	1.70	22.65	2.19	2.84	2.05	2.43	0.47	0.46	0.23	7.17	4.29
1-Nonene	0.11	0.12	0.09	0.43	0.15	4.71	1.32	0.18	0.06	0.06	0.01	0.10	0.04	0.65	0.42
1-Decene	1.11	1.73	1.59	0.36	1.38	1.83	1.24	1.80	0.56	0.65	2.80	0.66	1.26	20.58	12.99
$\sum_{6}$Alkenes	5.77	18.56	3.95	3.52	6.40	34.68	11.00	9.61	8.78	5.47	8.74	5.79	2.75	30.37	20.18
Benzene	0.28	0.31	0.41	0.04	0.19	0.35	0.11	0.22	0.12	0.18	0.27	0.17	0.13	0.51	0.43
Toluene	7.54	15.56	3.99	1.81	20.70	32.39	19.60	11.08	8.67	20.82	8.39	1.67	2.32	14.44	10.73
Ethylbenzene	1.93	0.47	1.19	0.09	0.33	0.33	0.29	0.47	0.38	0.34	0.64	0.38	0.16	5.66	3.36
m+p-Xylene	5.14	1.49	3.19	0.26	3.84	0.55	1.09	1.44	0.59	3.65	4.26	4.00	0.53	6.30	4.14
o-Xylene	2.23	0.66	1.33	0.12	0.48	0.36	0.42	0.64	0.27	0.56	0.99	0.61	0.20	4.16	2.57
Isopropylbenzene	0.04	0.05	0.06	0.01	0.03	0.05	0.16	0.11	0.08	0.03	0.06	0.03	0.03	5.38	3.06
n-Propylbenzene	0.12	0.11	0.10	0.03	0.10	0.11	0.14	0.15	0.06	0.12	0.21	0.14	0.07	8.48	4.85
1-Methyl-3-ethylbenzene	0.05	0.04	0.07	0.05	0.11	0.10	0.11	0.13	0.06	0.25	0.33	0.28	0.05	3.62	2.09
1-Methyl-4-ethylbenzene	0.04	0.04	0.05	0.02	0.12	0.06	0.09	0.11	0.06	0.10	0.16	0.12	0.08	1.49	0.92
1,3,5-Trimethylbenzene	0.05	0.05	0.05	0.03	0.11	0.08	0.11	0.13	0.05	0.15	0.25	0.16	0.07	1.54	0.95
1-Methyl-2-ethylbenzene	0.05	0.04	0.04	0.02	0.07	0.07	0.21	0.22	0.13	0.10	0.16	0.11	0.06	1.63	0.98
1,2,4-Trimethylbenzene	0.17	0.15	0.16	0.08	0.30	0.24	0.46	0.49	0.19	0.41	0.66	0.44	0.22	3.71	2.33
1,2,3-Trimethylbenzene	0.07	0.07	0.07	0.07	0.20	0.25	0.28	0.20	0.07	0.12	0.21	0.13	0.12	1.66	1.07
Indan	0.02	0.02	0.01	0.02	0.06	0.07	0.06	0.04	0.01	0.02	0.04	0.03	0.01	0.35	0.21
1,4-Diethylbenzene	0.02	0.05	0.05	0.04	0.11	0.13	0.15	0.12	0.03	0.06	0.11	0.07	0.06	1.14	0.71
n-Butylbenzene	0.02	0.00	0.05	0.03	0.09	0.09	0.02	0.03	0.01	0.03	0.07	0.04	0.06	1.35	0.83
1,2-Diethylbenzene	0.00	0.01	0.00	0.01	0.01	0.01	0.02	0.01	0.00	0.00	0.01	0.00	0.01	0.08	0.05
1,2,4,5-Tetramethylbenzene	0.03	0.03	0.02	0.03	0.08	0.08	0.10	0.06	0.02	0.03	0.05	0.04	0.03	0.46	0.30
1,2,3,5-Tetramethylbenzene	0.02	0.02	0.02	0.02	0.08	0.08	0.12	0.06	0.02	0.03	0.05	0.03	0.02	0.62	0.37
Naphthalene	0.31	0.10	0.07	0.04	0.31	0.10	0.93	0.24	0.29	0.84	0.08	2.04	0.06	2.69	1.58
Pentamethylbenzene	0.00	0.00	0.00	0.00	0.00	0.00	0.03	0.00	0.00	0.00	0.00	0.00	0.00	0.00	0.00
2-Methylnaphthalene	0.02	0.02	0.02	0.02	0.03	0.03	0.24	0.09	0.02	0.03	0.04	0.02	0.03	0.60	0.37
1-Methylnaphthalene	0.01	0.01	0.01	0.01	0.02	0.02	0.13	0.07	0.01	0.02	0.02	0.01	0.02	0.35	0.22
$\sum_{23}$Aromatics	18.15	19.29	10.96	2.85	27.35	35.53	24.78	16.17	11.14	27.90	17.06	10.52	4.39	66.10	42.12
Propionaldehyde	0.03	0.14	0.00	0.11	0.00	0.00	0.09	0.51	1.24	2.28	0.14	0.00	0.13	0.94	0.68
Isobutyraldehyde	8.70	1.32	1.32	0.97	11.87	3.37	0.51	32.48	2.09	5.03	0.34	2.41	1.39	1.35	2.32
Butyraldehyde	0.05	0.02	0.03	0.02	0.10	0.06	0.02	0.05	0.04	0.00	0.03	0.02	0.03	0.09	0.08
Isovaleraldehyde	0.00	0.00	0.01	0.02	0.01	0.01	0.01	0.07	0.01	0.00	0.00	0.00	0.00	0.15	0.09
n-Hexanal	1.65	1.05	2.01	0.81	1.91	1.47	0.64	0.90	0.43	0.27	0.95	0.46	0.22	21.73	12.48
n-Heptanal	0.55	0.68	0.67	0.45	0.70	0.25	0.46	0.38	0.17	0.20	0.56	0.12	0.10	8.86	5.09
Benzaldehyde	1.51	1.26	1.12	2.27	2.41	3.17	0.76	1.35	0.69	0.76	1.12	0.56	0.58	22.27	13.18
Octanal	0.49	0.64	0.60	1.39	1.78	2.58	3.92	1.94	0.35	0.44	0.67	0.31	0.15	15.66	8.98
Nonanal	2.98	4.35	3.48	5.04	5.81	11.96	1.86	3.02	1.48	2.40	4.38	2.06	1.05	0.40	1.41
Decanal	4.52	3.04	2.22	8.04	7.63	6.53	1.66	4.18	1.03	4.82	3.84	2.02	1.39	0.41	1.80
Undecanal	1.16	0.62	0.76	1.30	2.61	2.67	1.41	1.20	0.83	1.35	2.67	0.95	0.92	1.21	1.72
Dodecanal	1.40	1.81	0.73	1.37	1.75	1.51	1.21	1.64	0.55	1.45	1.52	0.64	0.82	0.39	1.15
$\sum_{12}$Aldehydes	23.04	14.92	12.95	21.79	36.58	33.57	13.54	46.24	9.39	19.01	16.21	9.54	7.79	72.09	49.32
Formic acid	0.61	0.49	2.66	2.21	7.79	15.48	10.11	7.36	3.33	2.17	0.14	12.29	2.04	9.06	7.39
Acetic acid	0.00	0.00	0.00	0.04	1.72	7.97	0.08	0.32	3.74	0.30	0.04	0.04	0.64	4.22	3.10
Propionic acid	0.13	0.00	0.00	0.03	0.00	0.00	0.04	0.08	0.05	0.00	0.08	0.00	0.00	0.00	0.00
Isobutyric acid	11.46	11.39	11.33	0.26	0.35	0.64	0.39	0.69	0.28	0.13	0.05	0.12	0.03	0.00	0.04
Hexanoic acid	0.53	0.42	3.38	0.87	1.24	2.36	2.54	0.63	0.77	1.94	1.96	0.56	0.86	0.00	0.97
$\sum_{5}$Acids	12.73	12.31	17.37	3.41	11.09	26.45	13.13	9.06	8.22	4.54	2.27	13.01	3.76	13.04	11.56
Acetone	10.08	10.59	28.44	4.56	9.87	11.65	12.74	12.55	12.50	5.98	41.64	4.07	13.86	9.21	20.77
2-Hexanone	0.27	0.21	0.21	1.39	3.39	2.36	0.27	0.22	0.75	0.11	0.81	0.15	0.10	5.17	3.01
$\sum_{2}$Ketones	10.35	10.80	28.65	5.95	13.26	14.00	13.11	12.90	13.02	6.09	42.45	4.21	14.33	13.86	23.92
$\sum_{56}$VOCs	298.30	225.80	168.38	79.52	221.99	250.89	128.37	162.75	129.11	198.94	236.02	117.96	51.97	341.97	250.82

(continued)

INDOOR SAMPLES															
	WARM PERIOD														
	U1			U2			U3			P			AIRP		
VOCs	25/7/2023	26/7/2023	27/7/2023	25/7/2023	26/7/2023	27/7/2023	25/7/2023	26/7/2023	27/7/2023	25/7/2023	26/7/2023	27/7/2023	25/7/2023	26/7/2023	27/7/2023
Pentane	15.35	4.07	19.14	9.11	1.17	1.63	1.62	9.10	7.02	7.10	33.70	2.17	2.18	1.28	3.17
Hexane	188.13	92.86	140.29	112.84	55.00	203.63	64.26	36.86	129.99	185.21	170.04	218.03	79.34	111.73	152.10
Heptane	17.62	1.12	1.55	1.64	0.12	26.86	2.06	3.70	8.05	2.44	2.57	6.75	11.97	26.62	28.44
Octane	0.58	0.56	0.58	0.45	0.18	0.17	0.98	3.28	2.31	0.94	30.03	0.80	6.75	14.70	15.87
Nonane	69.56	5.18	6.31	4.94	1.72	3.89	0.86	3.37	13.08	2.02	6.05	28.02	7.20	9.42	13.40
Decane	2.86	0.27	5.31	2.68	2.42	2.42	3.16	2.86	3.21	2.47	2.82	4.75	3.99	5.92	7.82
Undecane	0.60	0.45	1.82	1.58	1.56	1.57	2.24	3.38	3.07	2.59	2.02	3.41	6.24	8.75	11.94
Dodecane	0.76	0.52	0.98	0.73	0.83	0.58	2.54	0.81	0.45	1.48	1.11	2.36	3.70	3.19	5.96
$\sum_{8}$Alkanes	274.88	115.65	181.58	133.96	63.01	240.75	77.72	63.35	167.16	203.44	253.66	261.77	121.61	181.29	238.79
1-Pentene	4.38	1.06	5.85	0.73	0.42	0.63	0.38	0.74	1.14	5.46	0.98	1.56	0.72	0.31	0.98
1-Hexene	5.71	0.48	22.96	3.00	3.15	5.07	0.60	10.61	1.28	16.27	0.51	1.36	1.72	5.59	5.08
1-Heptene	4.41	1.78	1.57	10.75	0.24	1.75	0.25	0.13	0.42	4.79	3.16	9.45	0.73	1.32	1.57
1-Octene	1.35	0.22	0.41	0.53	0.14	0.21	0.44	0.62	0.52	0.13	1.77	0.97	0.38	0.05	0.45
1-Nonene	0.06	0.03	0.03	0.40	0.07	0.08	0.09	0.05	0.13	0.26	1.03	0.05	0.52	0.04	0.61
1-Decene	0.69	0.31	2.30	2.51	2.47	2.41	0.27	1.17	0.37	1.24	1.02	2.69	2.15	0.89	2.92
$\sum_{6}$Alkenes	16.61	3.88	33.11	17.93	6.49	10.14	2.03	13.32	3.84	34.09	7.35	11.26	6.44	7.89	11.68
Benzene	0.13	0.06	0.96	0.32	0.02	0.05	0.02	0.21	0.16	0.13	0.07	0.33	0.04	0.10	0.10
Toluene	25.10	5.57	9.00	8.82	3.39	4.83	9.40	9.79	10.94	15.21	18.38	17.96	1.75	8.09	6.51
Ethylbenzene	0.40	0.39	0.56	0.10	0.04	0.05	0.11	0.39	0.18	1.24	0.86	1.71	0.16	0.13	0.25
m+p-Xylene	0.12	0.10	0.62	0.41	0.15	0.15	0.27	1.02	0.43	3.38	2.79	4.12	0.68	0.39	0.98
o-Xylene	0.62	0.73	0.81	0.18	0.13	0.09	0.14	0.46	0.23	1.35	1.21	1.97	0.24	0.16	0.37
Isopropylbenzene	0.03	0.03	0.06	0.02	0.04	0.03	0.04	0.08	0.08	0.17	0.04	0.07	0.03	0.03	0.05
n-Propylbenzene	0.18	0.18	0.24	0.13	0.13	0.11	0.04	0.14	0.08	0.53	0.14	0.20	0.07	0.07	0.12
1-Methyl-3-ethylbenzene	0.29	0.28	0.37	0.19	0.19	0.16	0.04	0.19	0.06	0.57	0.24	0.35	0.08	0.04	0.12
1-Methyl-4-ethylbenzene	0.13	0.12	0.18	0.10	0.08	0.07	0.02	0.08	0.05	0.27	0.11	0.15	0.04	0.04	0.06
1,3,5-Trimethylbenzene	0.17	0.16	0.24	0.12	0.11	0.10	0.02	0.11	0.04	0.34	0.15	0.19	0.06	0.05	0.09
1-Methyl-2-ethylbenzene	0.12	0.11	0.18	0.12	0.11	0.10	0.02	0.09	0.03	0.26	0.11	0.15	0.05	0.05	0.08
1,2,4-Trimethylbenzene	0.45	0.43	0.65	0.36	0.35	0.30	0.07	0.38	0.10	0.99	0.44	0.60	0.17	0.18	0.29
1,2,3-Trimethylbenzene	0.13	0.13	0.23	0.17	0.16	0.14	0.03	0.16	0.04	0.33	0.15	0.19	0.11	0.11	0.18
Indan	0.03	0.03	0.04	0.05	0.05	0.05	0.01	0.03	0.01	0.07	0.04	0.04	0.01	0.03	0.03
1,4-Diethylbenzene	0.08	0.06	0.13	0.11	0.09	0.11	0.02	0.07	0.03	0.20	0.08	0.11	0.03	0.08	0.08
n-Butylbenzene	0.00	0.01	0.02	0.05	0.06	0.07	0.01	0.06	0.02	0.08	0.04	0.00	0.01	0.15	0.09
1,2-Diethylbenzene	0.00	0.00	0.01	0.02	0.18	0.02	0.01	0.01	0.00	0.01	0.01	0.01	0.00	0.01	0.01
1,2,4,5-Tetramethylbenzene	0.05	0.04	0.07	0.04	0.04	0.04	0.01	0.09	0.02	0.09	0.04	0.05	0.02	0.04	0.04
1,2,3,5-Tetramethylbenzene	0.04	0.03	0.06	0.03	0.03	0.02	0.01	0.06	0.01	0.09	0.03	0.05	0.01	0.02	0.02
Naphthalene	0.08	0.06	0.08	0.06	0.11	0.06	0.03	0.13	0.03	0.21	0.09	0.09	0.05	0.09	0.10
Pentamethylbenzene	0.00	0.00	0.00	0.01	0.01	0.00	0.00	0.00	0.00	0.01	0.01	0.00	0.00	0.00	0.00
2-Methylnaphthalene	0.03	0.02	0.03	0.04	0.09	0.05	0.01	0.04	0.02	0.07	0.04	0.04	0.02	0.05	0.05
1-Methylnaphthalene	0.02	0.01	0.02	0.02	0.06	0.03	0.01	0.03	0.01	0.04	0.03	0.02	0.01	0.03	0.04
$\sum_{23}$Aromatics	28.19	8.54	14.59	11.45	5.62	6.61	10.33	13.60	12.54	26.21	24.25	28.61	3.76	9.78	9.73
Propionaldehyde	0.03	2.02	0.47	0.11	0.04	0.04	0.03	0.00	0.04	0.43	2.24	0.12	0.01	0.96	0.55
Isobutyraldehyde	0.21	1.12	31.32	0.00	0.88	57.27	0.21	8.53	19.25	11.60	11.70	12.58	0.69	0.00	0.78
Butyraldehyde	0.03	0.01	2.18	0.01	0.00	0.00	0.04	0.01	0.02	0.06	0.02	0.03	0.03	0.00	0.03
Isovaleraldehyde	0.16	0.22	0.16	0.92	0.01	0.00	0.01	0.01	0.01	0.04	0.02	0.21	0.05	0.00	0.05
n-Hexanal	0.16	0.11	0.24	0.29	0.21	0.20	0.43	1.00	0.70	0.89	0.01	0.84	0.72	0.56	1.12
n-Heptanal	0.19	0.26	0.07	0.34	0.26	0.20	0.31	0.34	0.13	0.45	0.31	0.74	0.67	0.44	1.00
Benzaldehyde	1.21	0.94	0.78	1.54	1.13	0.71	0.85	2.36	1.04	2.74	1.23	1.60	2.80	1.09	3.77
Octanal	0.79	0.60	0.31	1.21	0.85	0.57	0.86	1.42	1.15	1.18	0.69	1.85	2.16	0.69	2.82
Nonanal	2.75	2.33	1.39	3.57	2.75	2.37	3.12	4.15	1.72	3.94	2.54	5.33	5.00	1.69	6.57
Decanal	3.18	2.22	1.54	5.91	4.76	3.49	3.75	4.51	2.11	5.06	3.30	10.02	1.01	6.07	4.55
Undecanal	1.29	1.18	0.68	1.56	1.73	0.91	1.67	2.17	1.67	2.03	1.21	2.67	2.50	3.15	4.59
Dodecanal	1.06	0.87	0.80	1.51	1.48	0.98	1.08	2.91	1.26	1.85	1.29	2.17	2.05	0.39	2.53
$\sum_{12}$Aldehydes	11.06	11.87	39.95	16.97	14.09	66.74	12.37	27.40	29.09	30.63	20.93	41.41	18.29	14.25	28.59
Formic acid	0.00	0.00	3.08	4.54	0.79	0.76	1.01	0.35	0.35	1.46	5.07	1.03	0.47	0.45	0.78
Acetic acid	10.66	0.84	0.00	0.00	21.60	0.00	0.03	0.26	0.27	14.99	2.29	64.99	0.10	0.03	0.13
Propionic acid	0.07	0.00	0.01	0.02	0.03	0.02	0.06	0.18	0.01	3.72	0.00	0.20	0.00	0.48	0.27
Isobutyric acid	0.59	0.50	0.96	0.12	0.01	0.02	0.13	0.95	0.12	0.01	0.29	0.10	0.49	0.00	0.56
Hexanoic acid	4.93	5.35	1.63	1.13	13.04	1.73	1.48	6.71	2.79	2.86	0.50	4.53	3.01	0.65	3.74
$\sum_{5}$Acids	16.25	6.69	5.68	5.81	35.46	2.53	2.70	8.44	3.55	23.01	8.07	70.95	4.15	1.50	5.51
Acetone	29.55	4.30	21.95	8.70	8.78	12.46	3.83	5.11	4.88	58.55	10.40	18.68	3.08	5.40	6.50
2-Hexanone	0.36	0.29	0.78	0.22	0.11	0.07	0.35	2.32	1.20	0.01	0.01	0.36	1.14	0.00	1.28
$\sum_{2}$Ketones	29.91	4.59	22.73	8.91	8.89	12.52	4.18	7.44	6.08	58.28	10.36	19.36	4.32	5.25	7.81
$\sum_{56}$VOCs	376.90	151.21	297.63	195.04	133.55	339.28	109.33	133.54	222.26	375.66	324.63	433.36	158.56	219.96	302.11

(continued)

	OUTDOOR SAMPLES																	
	COLD PERIOD																	
	U1			U2			U3			P			AIRP			UB		
	21/3/2023	22/3/2023	23/3/2023	21/3/2023	22/3/2023	23/3/2023	21/3/2023	22/3/2023	23/3/2023	21/3/2023	22/3/2023	23/3/2023	21/3/2023	22/3/2023	23/3/2023	21/3/2023	22/3/2023	23/3/2023
Pentane	4.76	5.85	4.44	0.81	0.87	8.21	1.30	3.11	1.25	0.52	0.63	1.65	0.37	1.06	0.88	4.01	2.81	7.92
Hexane	48.55	42.40	25.62	112.15	88.96	77.69	72.15	61.04	31.69	113.68	179.79	50.25	19.02	38.19	35.05	7.41	5.18	5.65
Heptane	1.09	0.41	0.16	0.32	1.04	0.79	2.42	0.77	0.76	13.39	0.61	1.59	0.33	0.16	0.30	0.21	0.11	0.64
Octane	0.59	0.97	0.51	0.28	0.33	0.32	1.51	0.89	0.73	1.39	0.49	0.13	2.66	0.14	1.72	3.11	0.20	1.08
Nonane	0.84	1.40	0.86	0.40	0.77	0.69	0.81	0.86	0.84	0.14	5.62	0.43	0.76	0.36	0.69	2.89	1.67	0.61
Decane	0.83	0.40	0.21	0.17	0.81	0.48	0.58	0.59	0.41	0.86	0.41	0.11	0.91	0.31	0.74	0.43	0.14	0.40
Undecane	0.51	0.22	0.14	0.26	0.50	0.26	0.46	0.19	0.11	0.20	0.28	0.10	0.30	0.38	0.42	0.23	0.08	0.71
Dodecane	0.71	0.39	0.34	0.20	0.33	0.26	0.48	0.13	0.10	0.15	0.51	0.08	0.32	0.51	0.51	0.21	0.10	0.36
$\sum_{8}$Alkanes	57.88	52.03	32.26	114.57	93.61	88.70	79.71	67.57	35.89	130.32	188.35	54.34	24.68	41.10	40.30	18.51	10.29	17.37
1-Pentene	0.65	8.95	1.56	0.23	2.77	1.45	6.22	6.42	6.90	0.91	1.20	6.13	1.24	3.64	2.99	0.81	0.54	5.69
1-Hexene	4.11	20.29	6.01	1.52	3.24	2.48	0.43	1.93	2.28	4.03	17.97	3.61	1.67	0.09	1.08	0.44	3.40	0.81
1-Heptene	6.99	3.01	2.50	0.17	8.73	6.88	1.67	4.43	0.99	32.79	14.69	1.52	0.00	0.05	0.03	0.06	0.83	0.21
1-Octene	3.34	1.72	0.16	0.56	26.68	12.04	5.76	4.14	3.87	3.19	0.32	0.40	0.69	0.00	0.42	0.14	0.51	1.18
1-Nonene	1.07	0.08	0.02	0.31	0.03	0.11	0.06	0.05	0.02	13.68	0.04	0.03	0.69	0.18	0.54	0.00	0.07	2.45
1-Decene	1.08	0.48	0.20	0.29	6.79	0.92	0.59	0.63	0.42	0.81	0.45	0.14	0.77	0.26	0.63	0.38	0.17	2.64
$\sum_{6}$Alkenes	17.24	34.54	10.45	3.07	48.23	23.88	14.73	17.60	14.47	55.42	34.67	11.82	5.06	4.22	5.68	1.84	5.53	12.98
Benzene	0.20	0.14	0.07	0.25	0.27	0.24	0.12	0.15	0.16	0.24	0.15	0.15	0.20	0.03	0.14	0.14	0.22	0.09
Toluene	24.19	10.01	5.08	1.66	27.66	12.63	28.94	18.20	13.75	24.10	15.45	5.13	2.92	4.23	4.38	5.02	3.49	2.30
Ethylbenzene	1.80	0.12	0.12	0.08	0.28	0.15	0.11	0.16	0.08	0.32	0.24	0.13	0.11	0.12	0.14	0.23	0.15	0.55
m+p-Xylene	0.40	0.58	0.43	0.25	0.38	0.34	0.32	0.66	0.31	1.03	0.84	0.21	0.20	0.35	0.34	0.76	0.74	1.82
o-Xylene	1.81	0.20	0.17	0.11	0.28	0.19	0.18	0.27	0.15	0.50	0.43	0.22	0.10	0.16	0.16	0.37	0.28	0.94
Isopropylbenzene	0.10	0.01	0.00	0.00	0.10	0.02	0.01	0.01	0.01	0.17	0.02	0.01	0.04	0.03	0.04	0.02	0.01	0.21
n-Propylbenzene	0.17	0.05	0.02	0.03	0.11	0.05	0.02	0.05	0.03	0.10	0.09	0.05	0.03	0.11	0.08	0.09	0.06	0.56
1-Methyl-3-ethylbenzene	0.06	0.03	0.03	0.04	0.09	0.06	0.03	0.07	0.04	0.21	0.14	0.10	0.03	0.17	0.13	0.16	0.05	0.87
1-Methyl-4-ethylbenzene	0.06	0.04	0.02	0.03	0.08	0.05	0.03	0.03	0.02	0.12	0.07	0.04	0.03	0.08	0.07	0.14	0.04	0.31
1,3,5-Trimethylbenzene	0.05	0.04	0.02	0.02	0.10	0.04	0.04	0.06	0.03	0.17	0.10	0.06	0.03	0.11	0.08	0.09	0.07	0.48
1-Methyl-2-ethylbenzene	0.03	0.03	0.02	0.02	0.06	0.07	0.03	0.04	0.02	0.09	0.08	0.05	0.02	0.08	0.06	0.06	0.05	0.45
1,2,4-Trimethylbenzene	0.12	0.09	0.05	0.07	0.25	0.11	0.12	0.14	0.08	0.35	0.31	0.17	0.06	0.37	0.27	0.27	0.24	1.61
1,2,3-Trimethylbenzene	0.05	0.03	0.02	0.03	0.09	0.05	0.04	0.04	0.03	0.11	0.10	0.05	0.02	0.11	0.08	0.08	0.07	0.68
Indan	0.01	0.01	0.01	0.01	0.02	0.01	0.01	0.01	0.01	0.04	0.02	0.01	0.01	0.03	0.02	0.02	0.01	0.14
1,4-Diethylbenzene	0.03	0.01	0.00	0.02	0.05	0.03	0.03	0.01	0.01	0.06	0.05	0.03	0.01	0.05	0.04	0.04	0.03	0.41
n-Butylbenzene	0.04	0.02	0.00	0.01	0.04	0.03	0.01	0.02	0.01	0.03	0.01	0.02	0.01	0.04	0.03	0.01	0.02	0.04
1,2-Diethylbenzene	0.00	0.00	0.00	0.01	0.003	0.02	0.00	0.00	0.00	0.03	0.00	0.00	0.00	0.03	0.02	0.00	0.00	0.03
1,2,4,5-Tetramethylbenzene	0.02	0.00	0.01	0.01	0.02	0.02	0.02	0.02	0.01	0.05	0.03	0.02	0.01	0.02	0.01	0.03	0.03	0.19
1,2,3,5-Tetramethylbenzene	0.01	0.01	0.01	0.01	0.01	0.01	0.02	0.01	0.01	0.03	0.03	0.02	0.01	0.02	0.01	0.03	0.03	0.25
Naphthalene	0.08	0.07	0.05	0.05	0.09	0.06	0.08	0.03	0.02	0.05	0.06	0.02	0.02	0.08	0.06	0.04	0.03	0.28
Pentamethylbenzene	0.00	0.01	0.00	0.001	0.001	0.001	0.00	0.00	0.00	0.00	0.00	0.00	0.00	0.00	0.00	0.00	0.00	0.05
2-Methylnaphthalene	0.03	0.03	0.02	0.02	0.03	0.02	0.03	0.01	0.01	0.02	0.04	0.02	0.00	0.03	0.02	0.02	0.02	0.28
1-Methylnaphthalene	0.02	0.02	0.01	0.02	0.01	0.01	0.02	0.01	0.01	0.02	0.02	0.01	0.00	0.02	0.01	0.01	0.01	0.15
$\sum_{23}$Aromatics	29.27	11.51	6.17	2.72	30.02	14.19	30.19	19.98	14.78	27.84	18.27	6.52	3.83	6.26	6.18	7.63	5.66	12.68
Propionaldehyde	0.29	0.15	0.00	0.09	4.55	3.79	0.00	0.11	0.00	0.73	0.22	0.03	1.15	0.33	0.91	0.09	0.01	0.03
Isobutyraldehyde	6.09	0.27	0.38	0.87	0.47	9.53	0.21	21.22	0.21	64.41	0.58	1.26	0.39	0.01	0.25	3.20	0.50	0.24
Butyraldehyde	0.00	0.01	0.00	0.09	0.10	0.00	0.03	0.06	0.03	0.03	0.01	0.01	0.11	0.02	0.08	0.06	0.02	0.01
Isovaleraldehyde	0.00	0.00	0.00	0.06	0.00	0.00	0.00	0.99	0.00	0.00	0.03	0.00	0.00	0.01	0.00	0.00	1.43	0.01
n-Hexanal	1.54	0.11	0.15	0.51	1.02	0.31	0.14	0.16	0.07	0.40	0.08	0.15	0.75	1.81	1.57	0.36	0.04	2.43
n-Heptanal	0.23	0.13	0.05	0.67	1.88	0.14	0.17	0.18	0.13	0.17	0.05	0.00	0.23	0.19	0.26	0.10	0.05	1.84
Benzaldehyde	1.02	0.55	0.44	1.30	1.86	1.37	1.23	0.57	0.48	0.48	0.51	0.28	0.40	0.80	0.73	0.41	0.37	0.84
Octanal	0.66	0.36	0.18	0.92	0.63	0.74	0.30	0.23	0.11	0.60	0.27	0.15	0.13	0.62	0.46	0.26	0.20	0.90
Nonanal	4.20	1.62	0.72	1.82	2.47	2.58	1.39	0.91	0.60	2.32	1.06	0.65	0.91	2.50	2.08	1.07	1.12	0.65
Decanal	1.76	3.71	2.30	5.11	1.90	5.22	1.95	1.52	0.99	3.22	2.03	1.19	1.10	1.84	1.80	2.08	2.55	0.50
Undecanal	1.20	1.23	0.89	1.23	0.90	2.14	0.29	0.65	0.42	0.83	0.86	0.48	0.41	2.02	1.49	0.42	0.32	0.96
Dodecanal	1.17	1.18	0.73	1.34	0.56	1.10	1.55	0.55	0.41	0.71	1.09	0.59	0.50	1.09	0.97	0.52	0.53	0.50
$\sum_{12}$Aldehydes	18.15	9.32	5.85	14.00	16.35	26.90	7.25	27.15	3.44	73.90	6.78	4.81	6.08	11.21	10.60	8.57	7.15	8.89
Formic acid	21.35	2.71	4.56	1.32	3.00	18.36	6.27	10.26	13.13	19.30	1.06	5.34	7.27	2.47	5.96	0.79	0.67	3.20
Acetic acid	1.05	1.09	1.12	3.32	0.31	0.87	0.34	28.42	2.35	2.61	0.37	9.31	4.49	0.13	2.83	0.20	0.37	0.29
Propionic acid	0.00	0.01	0.00	0.31	0.27	0.05	0.18	0.12	0.13	0.14	0.00	0.00	0.15	0.02	0.10	0.00	0.21	0.00
Isobutyric acid	0.06	0.21	0.03	0.20	0.09	0.22	0.16	0.11	0.11	0.19	1.47	0.02	0.10	0.29	0.24	0.22	0.01	0.98
Hexanoic acid	1.87	0.13	1.03	1.35	1.29	0.33	0.10	0.10	0.10	0.23	0.14	0.14	0.41	19.00	11.89	0.37	0.10	0.00
$\sum_{5}$Acids	24.33	4.15	6.75	6.50	4.96	19.82	7.06	39.01	15.81	22.46	3.04	14.81	12.41	21.90	21.02	1.59	1.36	4.48
Acetone	4.42	20.58	1.75	5.14	25.33	47.56	16.65	22.68	15.15	22.33	8.71	3.53	4.35	5.29	5.90	5.89	0.29	4.85
2-Hexanone	0.09	0.02	0.06	0.55	1.62	0.75	0.18	0.22	0.12	0.12	0.08	0.06	1.11	0.07	0.72	0.53	0.08	0.78
$\sum_{2}$Ketones	4.51	20.60	1.81	5.69	26.95	48.31	16.83	22.91	15.28	22.45	8.79	3.59	5.46	5.36	6.63	6.43	0.37	5.62
$\sum_{56}$VOCs	151.38	132.15	63.28	146.55	220.11	221.80	155.76	194.21	99.66	332.39	259.89	95.89	57.51	90.05	90.41	44.56	30.35	62.02

(continued)

	OUTDOOR SAMPLES																	
	WARM PERIOD																	
	U1			U2			U3			P			AIRP			UB		
	25/7/2023	26/7/2023	27/7/2023	25/7/2023	26/7/2023	27/7/2023	25/7/2023	26/7/2023	27/7/2023	25/7/2023	26/7/2023	27/7/2023	25/7/2023	26/7/2023	27/7/2023	25/7/2023	26/7/2023	27/7/2023
Pentane	5.44	1.32	0.70	4.52	5.34	4.53	5.36	2.82	3.29	12.95	25.45	6.61	0.20	3.37	2.23	2.39	0.80	0.39
Hexane	186.24	161.69	178.79	175.48	193.35	113.92	94.25	112.10	162.02	234.76	344.99	246.71	254.32	231.94	303.61	65.47	121.27	47.90
Heptane	17.26	1.53	5.35	2.74	5.42	3.76	0.80	1.16	1.79	65.66	1.80	8.36	4.76	11.63	10.23	2.33	1.38	1.27
Octane	1.08	0.38	0.24	0.22	0.60	0.15	0.09	0.13	0.21	6.90	28.43	1.71	0.09	3.50	2.24	1.71	1.95	1.75
Nonane	3.26	18.78	15.01	6.79	17.04	4.62	1.67	1.47	4.37	19.56	20.87	39.91	3.72	4.70	5.25	1.62	12.62	4.32
Decane	1.95	0.18	0.87	0.91	1.49	1.30	0.23	0.35	0.30	30.68	0.78	1.84	0.68	1.06	1.09	0.30	0.44	0.28
Undecane	0.52	0.30	0.38	0.37	1.07	0.42	0.07	0.09	0.12	26.10	0.40	0.93	0.26	0.66	0.57	0.17	0.15	0.11
Dodecane	0.67	0.31	0.27	1.43	0.61	0.58	0.08	0.16	0.12	27.33	0.41	0.66	0.27	0.54	0.50	0.13	0.16	0.09
$\sum_{8}$Alkanes	216.43	184.47	201.61	192.46	224.92	129.27	102.00	118.51	172.50	423.94	423.12	306.73	264.29	257.39	325.73	74.12	138.77	56.11
1-Pentene	1.55	0.37	0.18	2.44	2.11	5.31	1.59	1.46	1.81	2.85	6.19	3.86	1.27	0.98	1.41	1.11	0.24	0.10
1-Hexene	0.68	0.38	0.67	21.44	19.51	19.45	0.34	0.25	0.41	81.18	77.29	25.83	25.92	0.13	16.26	0.54	1.65	0.32
1-Heptene	5.94	1.85	1.91	0.20	1.59	0.09	1.45	1.01	1.35	7.09	2.06	3.35	1.16	2.24	2.12	1.66	1.87	1.66
1-Octene	0.27	0.80	0.47	0.45	0.76	0.41	0.13	0.16	0.15	10.76	10.12	10.19	0.36	0.15	0.32	1.24	1.80	1.48
1-Nonene	0.08	0.12	0.38	0.02	0.05	0.04	0.06	0.07	0.17	3.42	2.04	1.90	0.03	0.17	0.12	0.06	0.37	0.17
1-Decene	1.44	0.37	0.60	0.72	1.15	0.74	0.11	0.22	0.15	26.13	1.73	2.53	0.00	0.65	0.40	0.23	0.29	0.23
$\sum_{6}$Alkenes	9.95	3.89	4.21	25.26	25.17	26.04	3.64	3.17	4.07	131.43	99.43	47.65	28.73	4.31	20.63	4.84	6.22	3.95
Benzene	0.20	0.06	0.04	0.11	0.13	0.09	0.19	0.13	0.18	2.00	0.18	0.38	0.05	0.09	0.09	0.15	0.10	0.12
Toluene	34.78	17.62	12.46	21.81	25.28	13.29	4.61	7.57	12.61	21.46	9.72	22.70	38.62	1.67	25.15	4.60	8.83	14.40
Ethylbenzene	0.11	0.34	0.15	0.11	0.12	0.13	0.05	0.09	0.08	0.13	0.60	1.20	0.02	0.05	0.04	0.09	0.08	0.08
m+p-Xylene	0.22	1.08	0.60	0.32	0.39	0.37	0.15	0.26	0.25	5.99	1.66	3.00	0.07	0.17	0.14	0.34	0.37	0.30
o-Xylene	0.13	0.56	0.27	0.14	0.18	0.17	0.07	0.12	0.12	5.31	0.95	1.55	0.04	0.08	0.07	0.15	0.13	0.13
Isopropylbenzene	0.03	0.03	0.02	0.02	0.02	0.02	0.01	0.03	0.01	3.96	0.05	0.05	0.00	0.00	0.00	0.00	0.01	0.03
n-Propylbenzene	0.07	0.14	0.09	0.05	0.06	0.06	0.02	0.05	0.04	6.99	0.25	0.12	0.02	0.03	0.03	0.04	0.04	0.03
1-Methyl-3-ethylbenzene	0.06	0.25	0.15	0.09	0.11	0.08	0.03	0.05	0.04	4.65	0.37	0.12	0.06	0.04	0.06	0.04	0.05	0.03
1-Methyl-4-ethylbenzene	0.04	0.20	0.06	0.07	0.08	0.07	0.02	0.03	0.04	2.19	0.22	0.13	0.05	0.02	0.04	0.03	0.04	0.03
1,3,5-Trimethylbenzene	0.06	0.16	0.09	0.06	0.07	0.05	0.02	0.04	0.03	2.65	0.24	0.11	0.03	0.03	0.04	0.04	0.04	0.04
1-Methyl-2-ethylbenzene	0.04	0.11	0.06	0.04	0.07	0.05	0.01	0.03	0.02	2.52	0.17	0.07	0.06	0.02	0.05	0.02	0.02	0.02
1,2,4-Trimethylbenzene	0.17	0.43	0.24	0.15	0.28	0.17	0.04	0.09	0.08	0.14	0.69	0.27	0.07	0.08	0.09	0.09	0.08	0.08
1,2,3-Trimethylbenzene	0.07	0.13	0.10	0.06	0.29	0.07	0.01	0.03	0.02	2.74	0.19	0.10	0.02	0.00	0.01	0.03	0.03	0.02
Indan	0.02	0.03	0.02	0.01	0.04	0.01	0.00	0.01	0.01	0.54	0.05	0.02	0.01	0.01	0.01	0.01	0.01	0.01
1,4-Diethylbenzene	0.05	0.02	0.05	0.04	0.20	0.04	0.01	0.02	0.01	2.50	0.12	0.05	0.02	0.03	0.03	0.02	0.01	0.01
n-Butylbenzene	0.00	0.04	0.00	0.01	0.05	0.02	0.01	0.03	0.02	0.74	0.01	0.05	0.02	0.01	0.02	0.01	0.01	0.01
1,2-Diethylbenzene	0.00	0.00	0.00	0.00	0.01	0.00	0.03	0.16	0.03	0.15	0.01	0.00	0.01	0.00	0.01	0.00	0.00	0.00
1,2,4,5-Tetramethylbenzene	0.03	0.04	0.03	0.02	0.08	0.02	0.01	0.01	0.01	1.77	0.06	0.01	0.01	0.01	0.02	0.01	0.01	0.01
1,2,3,5-Tetramethylbenzene	0.02	0.04	0.01	0.02	0.09	0.02	0.00	0.01	0.01	1.87	0.06	0.02	0.01	0.02	0.02	0.01	0.01	0.01
Naphthalene	0.09	0.05	0.05	0.10	0.37	0.08	0.02	0.04	0.02	3.51	0.07	0.05	0.02	0.02	0.02	0.02	0.02	0.01
Pentamethylbenzene	0.00	0.00	0.00	0.00	0.01	0.00	0.00	0.01	0.00	0.31	0.01	0.00	0.00	0.00	0.00	0.00	0.00	0.00
2-Methylnaphthalene	0.03	0.03	0.03	0.02	0.06	0.04	0.02	0.06	0.02	2.63	0.04	0.02	0.01	0.02	0.02	0.03	0.01	0.01
1-Methylnaphthalene	0.02	0.02	0.02	0.01	0.04	0.02	0.01	0.04	0.01	1.65	0.02	0.01	0.01	0.01	0.01	0.01	0.01	0.00
$\sum_{23}$Aromatics	36.25	21.38	14.53	23.26	28.02	14.84	5.32	8.91	13.65	76.39	15.72	30.03	39.19	2.41	25.97	5.74	9.91	15.38
Propionaldehyde	1.13	0.05	0.06	0.13	0.04	0.00	0.06	0.05	0.08	4.38	3.92	4.03	0.13	0.16	0.18	0.00	0.07	2.49
Isobutyraldehyde	0.66	9.77	0.57	13.21	46.84	2.20	0.04	0.03	0.09	17.02	4.74	2.60	0.00	0.00	0.00	5.50	0.55	1.21
Butyraldehyde	0.05	0.01	0.04	0.04	0.06	0.03	0.03	0.03	0.11	0.14	0.09	0.05	0.00	0.00	0.00	0.01	0.02	0.44
Isovaleraldehyde	0.36	0.36	0.36	0.00	0.00	0.00	0.35	0.46	0.32	0.65	0.13	0.00	0.00	0.00	0.00	0.86	0.00	0.00
n-Hexanal	0.61	0.19	0.46	0.32	0.33	0.27	0.11	0.15	0.17	18.09	0.54	0.31	0.03	0.15	0.11	0.12	0.32	0.15
n-Heptanal	0.64	0.21	0.84	0.09	0.11	0.11	0.13	0.16	0.18	14.62	0.38	0.11	0.04	0.14	0.11	0.18	0.36	0.15
Benzaldehyde	1.69	1.14	3.09	0.89	1.68	1.18	0.85	1.10	1.00	0.81	1.81	1.82	0.50	0.74	0.77	1.02	1.20	0.48
Octanal	1.03	0.73	1.56	0.21	1.82	0.33	0.47	0.57	0.65	28.68	1.56	0.99	0.23	0.37	0.38	0.53	1.24	0.57
Nonanal	2.32	1.88	3.42	0.98	1.97	1.39	1.36	1.61	2.05	26.00	3.47	2.71	0.98	1.20	1.36	1.69	2.96	1.54
Decanal	7.13	6.70	9.04	2.92	2.61	4.20	2.77	3.30	3.62	45.43	5.52	4.24	1.54	1.79	2.08	0.12	4.88	2.81
Undecanal	1.01	0.89	1.82	1.61	1.04	1.07	0.80	1.30	1.09	20.25	1.70	1.52	1.28	0.86	1.34	1.34	1.22	0.75
Dodecanal	1.14	0.96	2.24	0.77	1.49	1.01	0.77	1.13	0.90	4.09	1.58	1.17	0.56	1.24	1.13	0.19	1.10	0.70
$\sum_{12}$Aldehydes	17.78	22.88	23.50	21.17	58.00	11.79	7.72	9.85	10.29	180.14	25.43	19.55	5.30	6.65	7.46	11.54	13.91	11.29
Formic acid	0.21	2.58	0.27	8.66	6.96	70.69	0.30	0.43	0.94	23.96	4.31	1.20	41.49	0.83	26.43	0.38	0.04	0.92
Acetic acid	9.25	13.85	2.14	8.99	8.49	6.44	56.89	20.00	17.90	14.23	7.48	6.93	11.98	0.62	7.87	0.00	0.00	0.02
Propionic acid	0.18	0.00	2.11	0.12	0.14	0.12	0.04	0.03	0.02	31.44	2.03	0.22	1.40	0.29	1.06	0.11	0.03	0.00
Isobutyric acid	0.42	0.16	0.79	0.27	0.31	0.27	0.03	0.11	0.04	32.88	0.91	0.90	0.00	0.02	0.01	0.04	0.02	0.06
Hexanoic acid	2.54	0.95	1.97	0.65	0.74	1.48	0.22	0.22	0.71	3.79	1.85	1.74	0.00	0.00	0.00	0.84	0.43	0.10
$\sum_{5}$Acids	12.60	17.54	7.27	18.69	16.62	79.00	57.34	20.80	19.73	106.30	16.58	10.98	54.88	1.76	35.37	1.36	0.52	1.11
Acetone	34.25	5.41	3.59	6.53	24.56	3.23	5.97	4.33	4.47	63.06	18.24	40.72	35.38	4.94	25.18	8.53	8.83	7.80
2-Hexanone	0.83	0.32	0.14	0.39	0.41	0.28	0.16	0.24	0.25	29.17	0.67	0.96	0.03	0.00	0.02	0.22	0.40	0.21
$\sum_{2}$Ketones	35.08	5.73	3.73	6.92	24.97	3.51	6.13	4.57	4.72	92.24	18.91	41.69	35.42	4.94	25.20	8.76	9.24	8.01
$\sum_{56}$VOCs	328.07	255.88	254.86	287.75	377.70	264.45	182.17	165.81	224.95	1010.44	599.19	456.63	427.81	277.46	440.35	106.36	178.57	95.85

Table S3b. Summary statistics for concentrations (μg m^{-3}) of the various chemical classes of VOCs detected in indoor and outdoor air in Rhodes

	INDOOR COLD PERIOD					INDOOR WARM PERIOD					OUTDOOR COLD PERIOD					OUTDOOR WARM PERIOD				
	Mean	Median	Min	Max	SD	Mean	Median	Min	Max	SD	Mean	Median	Min	Max	SD	Mean	Median	Min	Max	SD
	U1																			
$\sum_{23}$Aromatics	16.1	18.0	8.7	19.8	5.0	17.1	18.6	1.4	29.9	13.9	15.6	14.5	1.4	32.2	13.3	24.0	19.2	14.4	43.4	13.6
$\sum_{12}$Aldehydes	17.0	17.1	10.1	23.5	6.6	21.0	12.7	9.9	48.5	18.4	11.1	9.1	4.9	21.3	9.6	21.4	19.3	12.1	34.9	9.6
$\sum_{2}$Ketones	16.6	12.7	4.1	36.8	14.1	19.1	19.0	2.3	36.0	15.1	9.0	4.6	0.77	25.8	15.5	14.8	5.6	3.2	45.0	20.1
$\sum_{8}$Alkanes	158	164	62.4	241	76.5	191	192	42.0	336	131	47.4	49.7	19.4	70.7	86.2	201	205	156	236	34.5
$\sum_{6}$Alkenes	9.4	8.1	1.0	20.5	8.9	17.9	15.3	1.0	40.0	16.3	20.7	19.1	6.3	38.4	12.2	6.0	5.9	2.1	10.2	4.3
$\sum_{5}$Acids	14.1	4.3	0.6	47.4	22.4	9.5	7.1	4.9	19.0	6.4	11.7	6.2	3.9	30.8	9.1	12.5	12.6	5.5	19.2	5.6
$\sum_{56}$VOCs	231	249	126	300	82.5	275	312	64.0	413	155	116	132	40.1	158	101	280	263	233	360	55.3
	U2																			
$\sum_{23}$Aromatics	21.9	21.6	0.4	44.0	20.1	7.9	6.3	1.7	17.3	7.1	15.6	11.3	1.8	38.2	18.5	22.0	16.2	1.8	54.0	22.5
$\sum_{12}$Aldehydes	30.6	33.5	15.8	39.7	10.4	32.6	19.3	10.8	80.9	32.7	19.1	16.6	10.9	32.2	20.9	30.3	18.3	11.5	73.2	29.1
$\sum_{2}$Ketones	11.1	12.6	2.4	16.7	6.1	10.1	6.9	4.5	22.0	8.3	27.0	20.4	4.2	63.0	21.5	11.8	8.8	0.52	29.1	12.6
$\sum_{8}$Alkanes	92.0	111.2	11.3	134	56.0	146	151	22.7	260	101	99.0	111	31.2	142	66.2	182	177	123	251	57.9
$\sum_{6}$Alkenes	14.9	8.1	0.8	42.4	18.9	11.5	11.1	1.0	22.8	9.9	25.1	18.3	1.7	61.9	32.0	25.5	5.5	4.8	86.2	40.4
$\sum_{5}$Acids	13.7	9.8	2.2	32.9	13.5	14.6	6.0	1.2	45.2	20.5	10.4	6.1	4.5	24.9	30.5	38.1	29.5	5.1	88.3	39.5
$\sum_{56}$VOCs	184	221	32.9	261	103	223	222	85.9	360	120	196	223	63.4	275	102	310	313	231	382	74.2
	U3																			
$\sum_{23}$Aromatics	17.4	17.7	4.7	29.4	11.2	12.2	7.8	4.1	29.0	11.4	21.7	15.6	1.9	53.5	17.2	9.3	7.2	2.3	20.5	8.1
$\sum_{12}$Aldehydes	23.1	11.4	4.9	64.4	27.9	23.0	23.4	11.3	33.6	11.2	12.6	7.6	1.8	33.5	9.6	9.3	9.3	6.6	11.9	2.5
$\sum_{2}$Ketones	13.0	4.2	3.5	40.1	18.1	5.9	5.6	2.1	10.3	3.4	18.3	6.8	0.68	59.1	19.4	5.1	4.8	2.9	8.0	2.1
$\sum_{8}$Alkanes	66.7	61.7	29.5	114	35.5	103	74.8	57.7	204	68.2	61.1	62.2	11.8	108	68.5	131	108	66.6	242	78.0
$\sum_{6}$Alkenes	9.8	10.3	2.7	15.9	5.5	6.4	4.6	0.6	15.7	6.6	15.6	6.2	3.9	46.0	15.0	3.6	2.0	1.0	9.6	4.0
$\sum_{5}$Acids	10.1	9.6	5.7	15.7	4.7	4.9	4.0	2.0	9.6	3.4	20.6	14.7	5.2	47.8	26.1	32.6	27.1	3.6	72.7	33.8
$\sum_{56}$VOCs	140	127	100	207	50.7	155	130	105	255	68.1	150	138	37.1	287	84.0	191	169	140	285	65.2

	INDOOR COLD PERIOD					INDOOR WARM PERIOD					OUTDOOR COLD PERIOD					OUTDOOR WARM PERIOD				
	**Mean**	**Median**	**Min**	**Max**	**SD**	**Mean**	**Median**	**Min**	**Max**	**SD**	**Mean**	**Median**	**Min**	**Max**	**SD**	**Mean**	**Median**	**Min**	**Max**	**SD**
	**AIRP**																			
$\sum_{23}$Aromatics	37.5	42.1	4.4	66.1	31.1	7.8	9.7	3.8	9.8	3.5	5.4	6.2	3.8	6.3	1.4	22.5	26.0	2.4	39.2	18.6
$\sum_{12}$Aldehydes	43.1	49.3	7.8	72.1	32.6	20.4	18.3	14.2	28.6	7.4	9.3	10.6	6.1	11.2	2.8	6.5	6.6	5.3	7.5	1.1
$\sum_{2}$Ketones	17.4	14.3	13.9	23.9	5.7	5.8	5.2	4.3	7.8	1.8	5.8	5.5	5.4	6.6	0.7	21.9	25.2	4.9	35.4	15.5
$\sum_{8}$Alkanes	89.7	104	18.9	147	64.9	181	181	122	239	58.6	35.4	40.3	24.7	41.1	9.3	282	264	257	326	37.6
$\sum_{6}$Alkenes	17.8	20.2	2.8	30.4	14.0	8.7	7.9	6.4	11.7	2.7	5.0	5.1	4.2	5.7	0.7	17.9	20.6	4.3	28.7	12.4
$\sum_{5}$Acids	9.5	11.6	3.8	13.0	5.0	3.7	4.2	1.5	5.5	2.0	18.4	21.0	12.4	21.9	5.2	30.7	35.4	1.8	54.9	26.9
$\sum_{56}$VOCs	215	251	52.0	342	148	227	220	159	302	72.0	79.3	90.0	57.5	90.4	18.9	382	428	277	440	90.6
	**P**																			
$\sum_{23}$Aromatics	18.5	20.4	5.0	28.1	11.2	26.4	25.9	22.8	31.0	3.5	17.5	16.2	4.7	33.1	31.2	40.7	29.0	4.6	100	42.1
$\sum_{12}$Aldehydes	14.9	14.2	9.3	22.0	6.2	31.0	24.2	13.2	62.4	21.8	28.5	7.4	3.5	95.6	77.4	75.0	28.6	14.4	229	103
$\sum_{2}$Ketones	17.6	6.3	1.7	56.0	25.7	29.3	12.3	1.3	91.4	42.2	11.6	9.5	1.2	26.3	35.5	50.9	50.2	2.8	101	42.3
$\sum_{8}$Alkanes	120	117	73.4	173	51.1	240	251	168	288	56.0	124	108	45.1	236	170	385	405	245	483	118
$\sum_{6}$Alkenes	6.7	6.0	4.6	10.1	2.4	17.6	17.8	5.2	29.5	12.6	34.0	30.0	2.9	72.9	50.0	92.8	87.0	42.8	154.5	50.4
$\sum_{5}$Acids	6.6	4.3	0.7	17.1	7.3	34.0	20.6	0.6	94.2	42.0	13.4	11.1	2.3	29.2	42.8	44.6	21.9	3.8	130.9	58.8
$\sum_{56}$VOCs	184	174	116	274	77.2	378	364	332	451	53.1	229	210	81.9	416	354	689	555	447	1198	352
	**UB**																			
$\sum_{23}$Aromatics											8.7	7.6	5.7	12.7	3.6	10.3	8.6	5.7	18.5	6.0
$\sum_{12}$Aldehydes											8.2	8.6	7.1	8.9	0.9	12.2	12.9	6.8	16.3	3.9
$\sum_{2}$Ketones											4.1	5.6	0.37	6.4	3.3	8.7	3.8	2.5	24.6	10.6
$\sum_{8}$Alkanes											15.4	17.4	10.3	18.5	4.5	89.7	77.9	26.7	176	63.3
$\sum_{6}$Alkenes											6.8	5.5	1.8	13.0	5.7	5.0	3.1	1.0	12.9	5.4
$\sum_{5}$Acids											2.5	1.6	1.4	4.5	1.7	1.0	1.0	0.2	1.8	0.7
$\sum_{56}$VOCs											45.6	44.6	30.3	62.0	15.9	127	109	77.2	212	59.2

*Only compounds with frequencies of detection ≥ 50% are included

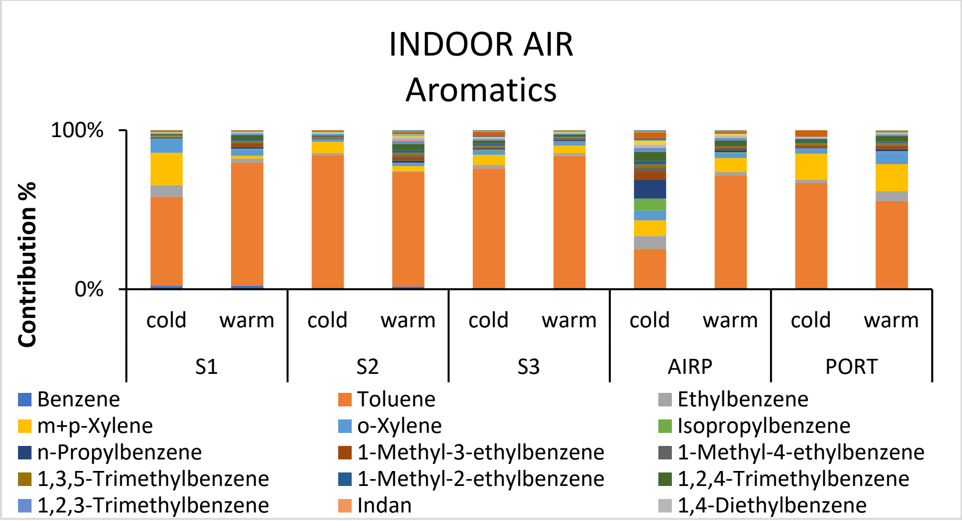
INDOOR AIR
Aromatics
100%
0%
Contribution %
cold
warm
cold
warm
cold
warm
cold
warm
cold
warm
S1
S2
S3
AIRP
PORT
Benzene
Toluene
Ethylbenzene
m+p-Xylene
o-Xylene
Isopropylbenzene
n-Propylbenzene
1-Methyl-3-ethylbenzene
1-Methyl-4-ethylbenzene
1,3,5-Trimethylbenzene
1-Methyl-2-ethylbenzene
1,2,4-Trimethylbenzene
1,2,3-Trimethylbenzene
Indan
1,4-Diethylbenzene

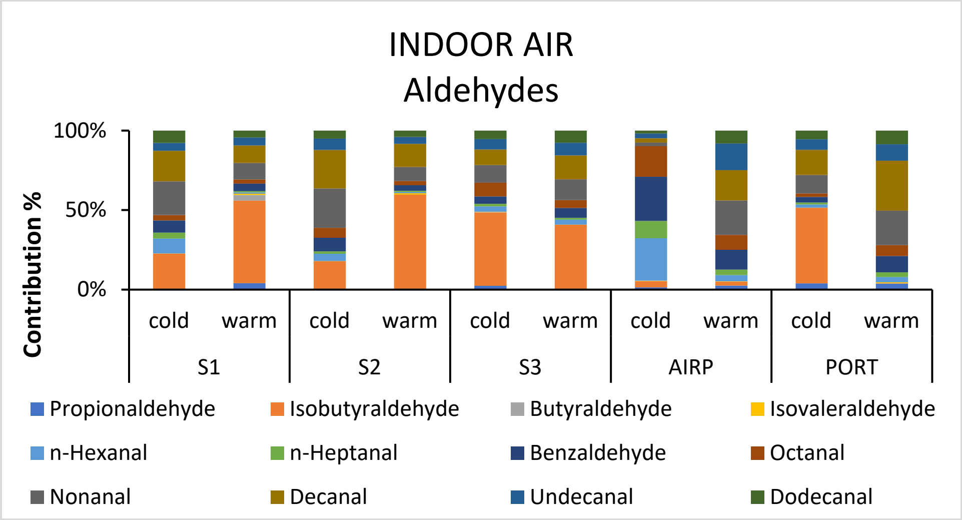
INDOOR AIR
Aldehydes
100%
50%
0%
Contribution %
cold
warm
cold
warm
cold
warm
cold
warm
cold
warm
S1
S2
S3
AIRP
PORT
Propionaldehyde
Isobutyraldehyde
Butyraldehyde
Isovaleraldehyde
n-Hexanal
n-Heptanal
Benzaldehyde
Octanal
Nonanal
Decanal
Undecanal
Dodecanal

(continued)

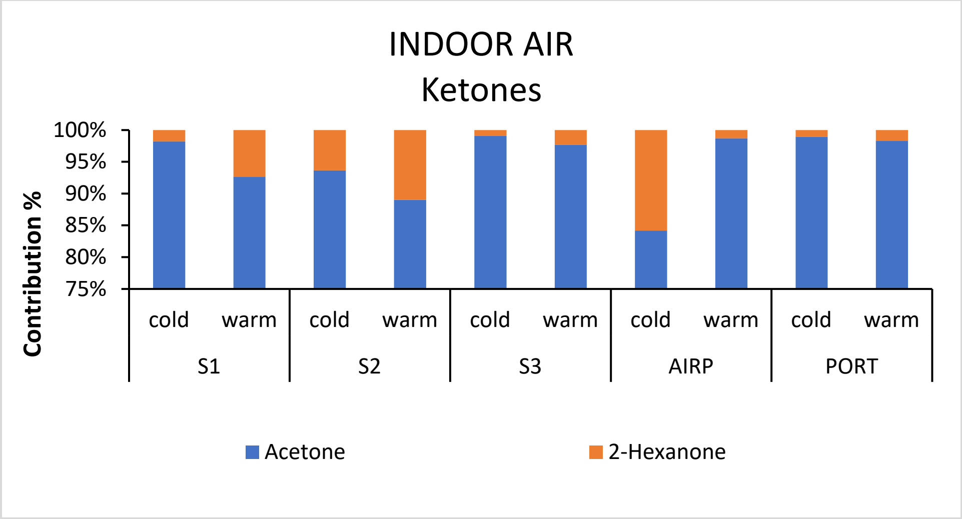
INDOOR AIR
Ketones
Contribution %
100%
95%
90%
85%
80%
75%
cold
warm
S1
S2
S3
AIRP
PORT
Acetone
2-Hexanone

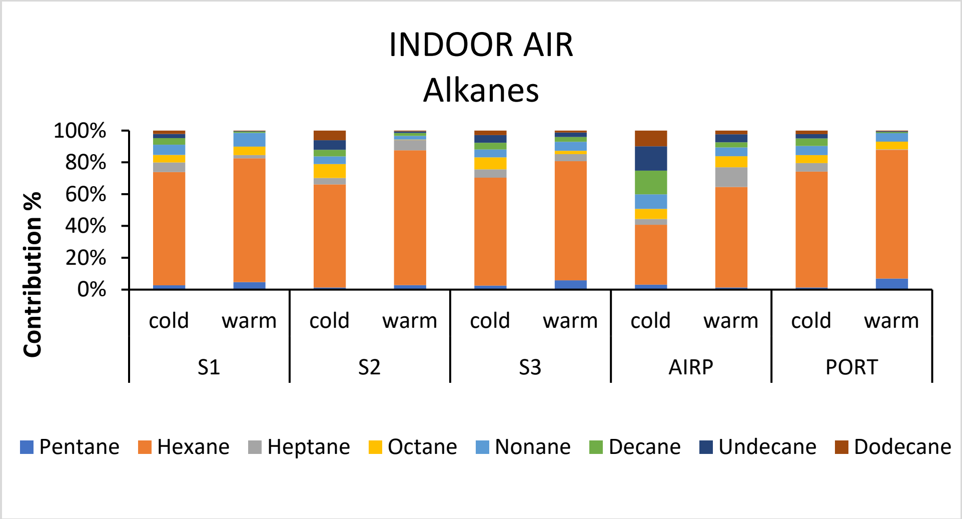
INDOOR AIR
Alkanes
Contribution %
100%
80%
60%
40%
20%
0%
cold
warm
S1
S2
S3
AIRP
PORT
Pentane
Hexane
Heptane
Octane
Nonane
Decane
Undecane
Dodecane

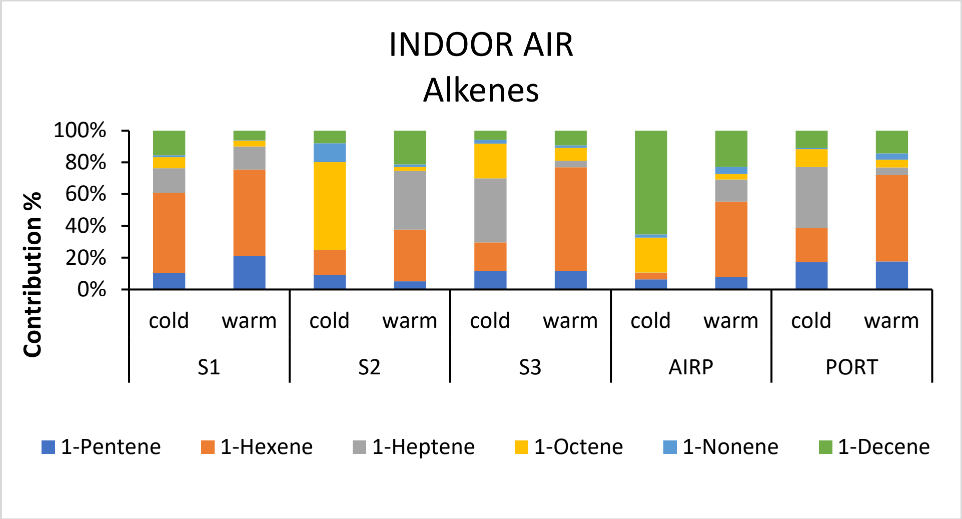
INDOOR AIR
Alkenes
Contribution %
100%
80%
60%
40%
20%
0%
cold
warm
S1
S2
S3
AIRP
PORT
1-Pentene
1-Hexene
1-Heptene
1-Octene
1-Nonene
1-Decene

(continued)

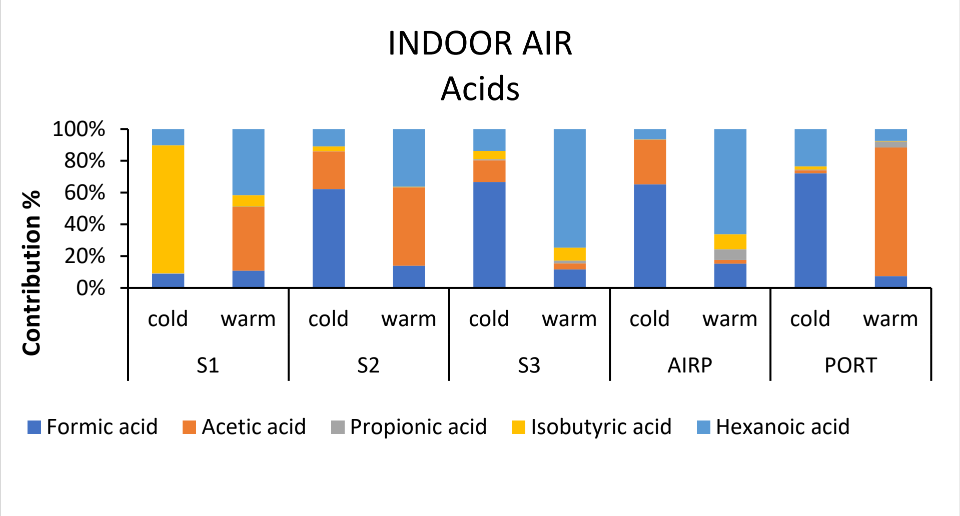
INDOOR AIR
Acids
100%
80%
60%
40%
20%
0%
Contribution %
cold
warm
S1
S2
S3
AIRP
PORT
Formic acid
Acetic acid
Propionic acid
Isobutyric acid
Hexanoic acid

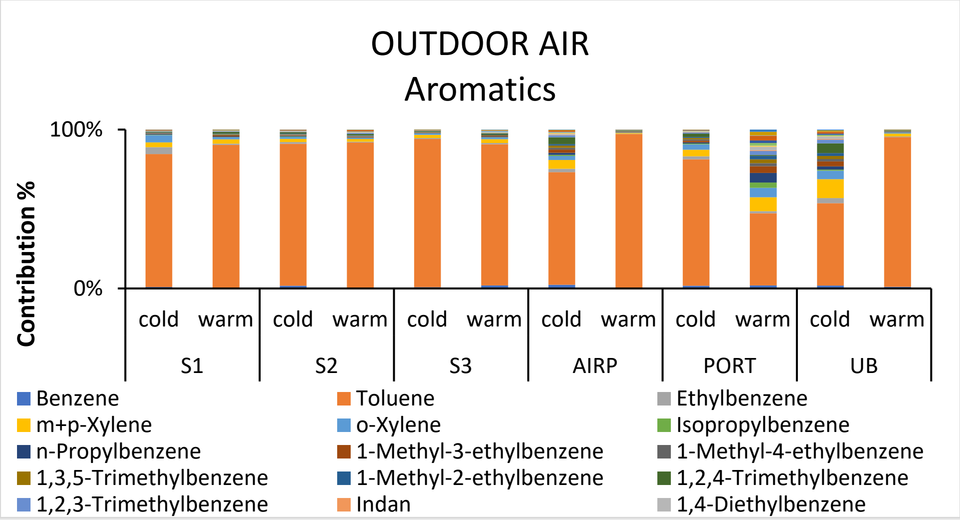
OUTDOOR AIR
Aromatics
100%
0%
Contribution %
cold
warm
S1
S2
S3
AIRP
PORT
UB
Benzene
Toluene
Ethylbenzene
m+p-Xylene
o-Xylene
Isopropylbenzene
n-Propylbenzene
1-Methyl-3-ethylbenzene
1-Methyl-4-ethylbenzene
1,3,5-Trimethylbenzene
1-Methyl-2-ethylbenzene
1,2,4-Trimethylbenzene
1,2,3-Trimethylbenzene
Indan
1,4-Diethylbenzene

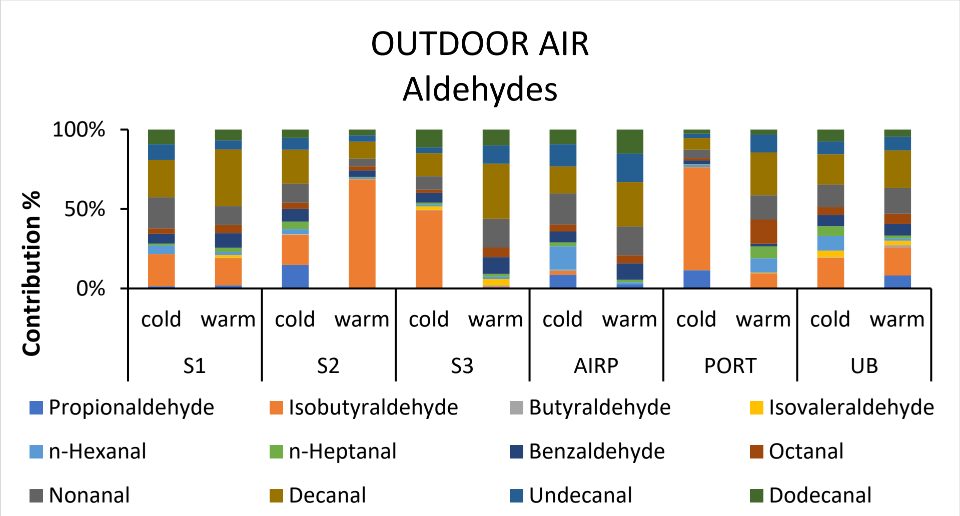
OUTDOOR AIR
Aldehydes
100%
50%
0%
Contribution %
cold
warm
S1
S2
S3
AIRP
PORT
UB
Propionaldehyde
Isobutyraldehyde
Butyraldehyde
Isovaleraldehyde
n-Hexanal
n-Heptanal
Benzaldehyde
Octanal
Nonanal
Decanal
Undecanal
Dodecanal

(continued)

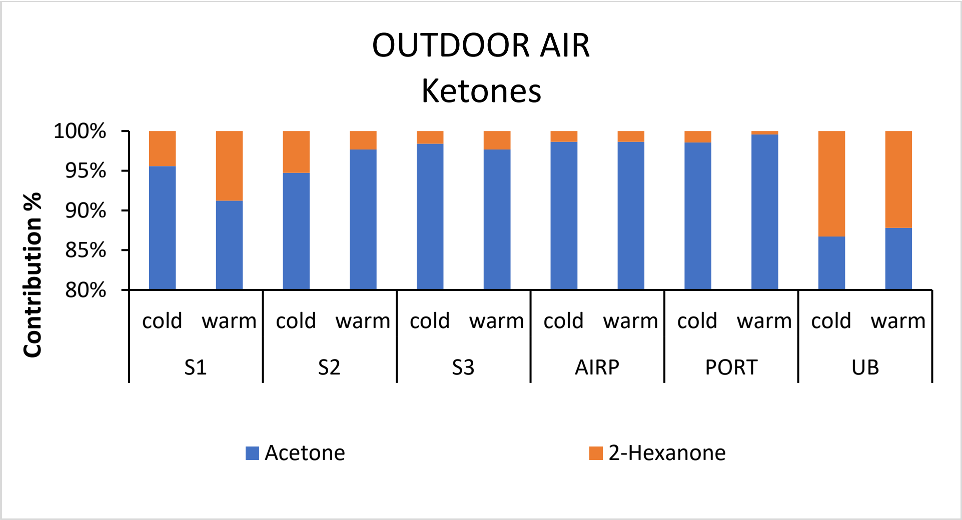
OUTDOOR AIR
Ketones
100%
95%
90%
85%
80%
Contribution %
cold
warm
S1
S2
S3
AIRP
PORT
UB
Acetone
2-Hexanone

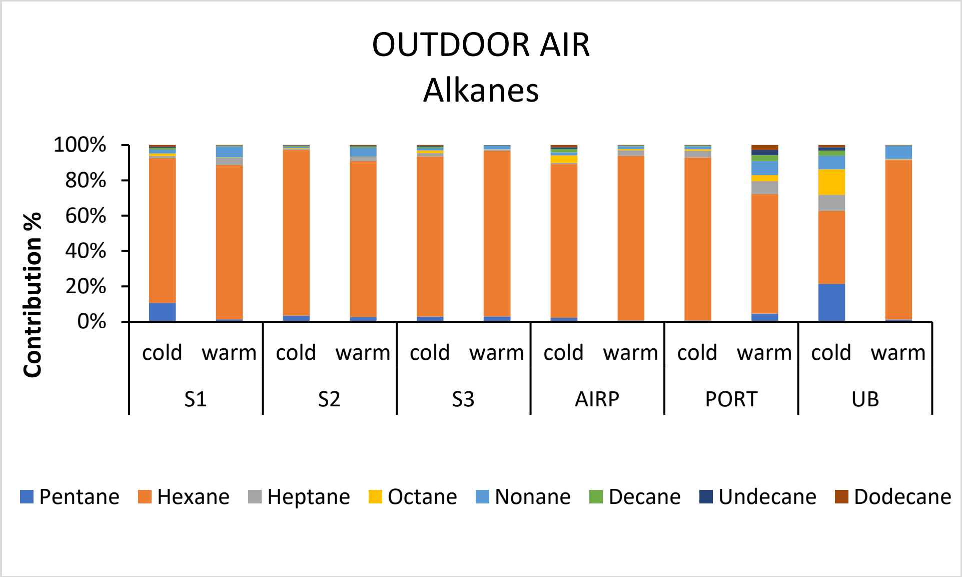
OUTDOOR AIR
Alkanes
100%
80%
60%
40%
20%
0%
Contribution %
cold
warm
S1
S2
S3
AIRP
PORT
UB
Pentane
Hexane
Heptane
Octane
Nonane
Decane
Undecane
Dodecane

(continued)

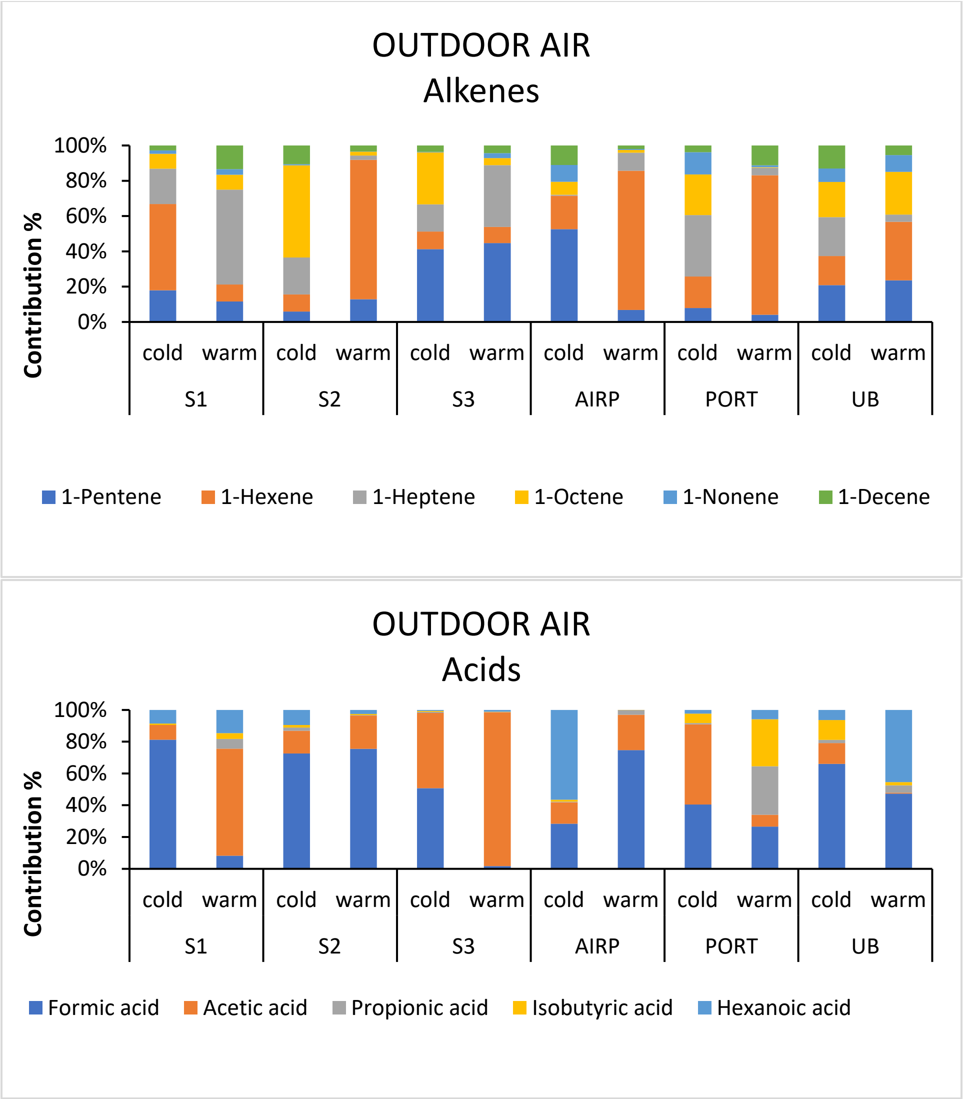

Figure S1. . Profiles of VOC categories in the indoor and outdoor air.

Table S4a: Spearman correlation coefficients between the chemical classes of outdoor VOCs and meteorological data

Correlations										
	**Aromatics**	**Aldehydes**	**Ketones**	**Alkanes**	**Alkenes**	**Acids**	**Temperature (^{O}C)**	**RH (%)**	**Wind speed (m s^{-1})**	**Wind direction**
**Aromatics**										
**Aldehydes**	0.829*									
**Ketones**		0.714*								
**Alkanes**										
**Alkenes**	0.967**			0.629*						
**Acids**		0.820*								
**Temperature (^{O}C)**				0.928**						
**RH (%)**		-0.866**	-0.714*				-0.899**			
**Wind speed (m s^{-1})**								-0.899**		
**Wind direction**								0.830*		

*. Correlation is significant at the 0.05 level (2-tailed). **. Correlation is significant at the 0.01 level (2-tailed).

Table S4b: Spearman correlation coefficients between the chemical classes of indoor VOCs and characteristics of indoor sampling sites and potential sources

	**Aromatics**	**Aldehydes**	**Ketones**	**Alkanes**	**Alkenes**	**Acids**	**Area (m2)**	**No. of furniture and EEEa**	**Ventilation**
**Aromatics**									
**Aldehydes**									
**Ketones**	.714*								
**Alkanes**	.668*								
**Alkenes**									
**Acids**									
Area (m^2)									
**No. of furniture and EEEa**	.697*	.738*							
**Ventilation**			.692*			.623*			1

Correlation is significant at the 0.05 level (2-tailed).**. Correlation is significant at the 0.01 level (2-tailed).

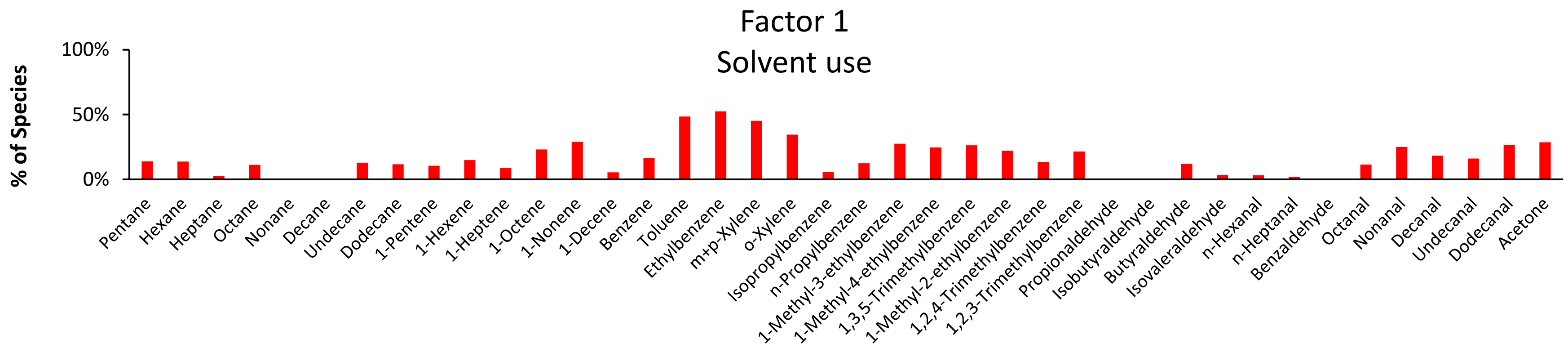
Factor 1
Solvent use
% of Species
100%
50%
0%
Pentane
Hexane
Heptane
Octane
Nonane
Decane
Undecane
Dodecane
1-Pentene
1-Hexene
1-Heptene
1-Octene
1-Nonene
1-Decene
Benzene
Toluene
Ethylbenzene
m+p-Xylene
o-Xylene
Isopropylbenzene
n-Propylbenzene
1-Methyl-3-ethylbenzene
1-Methyl-4-ethylbenzene
1,3,5-Trimethylbenzene
1-Methyl-2-ethylbenzene
1,2,4-Trimethylbenzene
1,2,3-Trimethylbenzene
Propionaldehyde
Isobutyraldehyde
Butyraldehyde
Isovaleraldehyde
n-Hexanal
n-Heptanal
Benzaldehyde
Octanal
Nonanal
Decanal
Undecanal
Dodecanal
Acetone

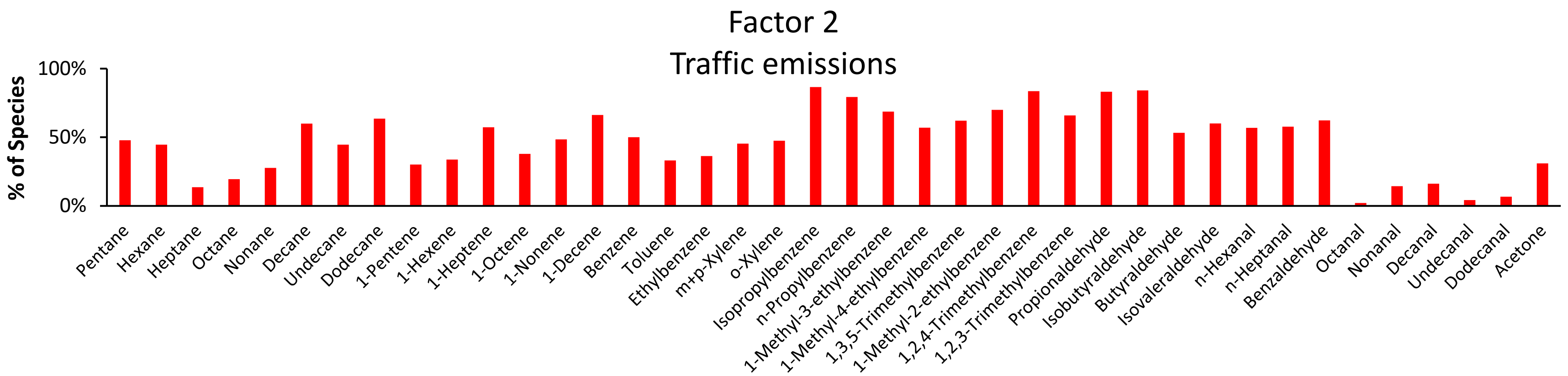
Factor 2
Traffic emissions
% of Species
100%
50%
0%
Pentane
Hexane
Heptane
Octane
Nonane
Decane
Undecane
Dodecane
1-Pentene
1-Hexene
1-Heptene
1-Octene
1-Nonene
1-Decene
Benzene
Toluene
Ethylbenzene
m+p-Xylene
o-Xylene
Isopropylbenzene
n-Propylbenzene
1-Methyl-3-ethylbenzene
1-Methyl-4-ethylbenzene
1,3,5-Trimethylbenzene
1-Methyl-2-ethylbenzene
1,2,4-Trimethylbenzene
1,2,3-Trimethylbenzene
Propionaldehyde
Isobutyraldehyde
Butyraldehyde
Isovaleraldehyde
n-Hexanal
n-Heptanal
Benzaldehyde
Octanal
Nonanal
Decanal
Undecanal
Dodecanal
Acetone

(continued)

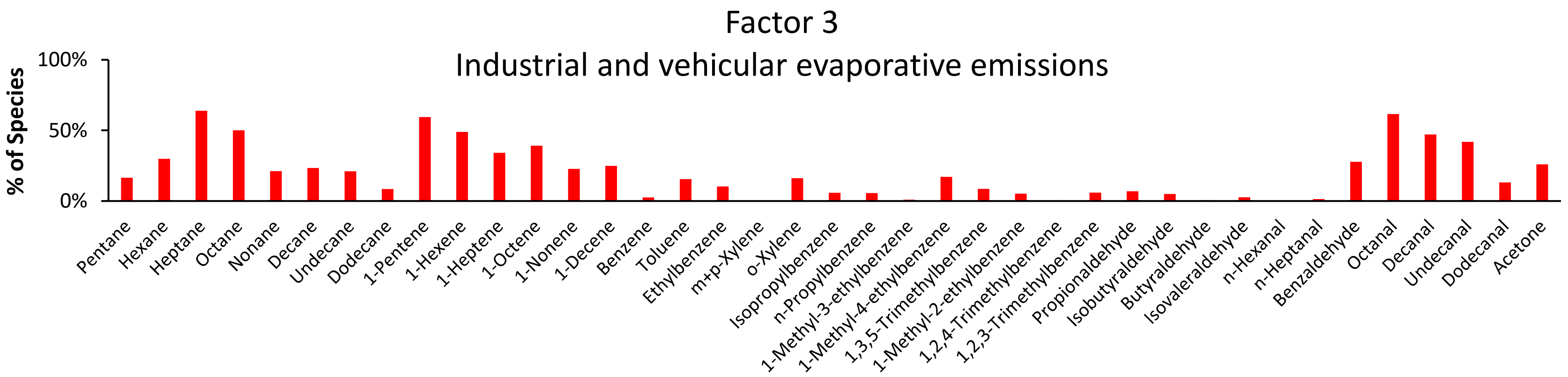

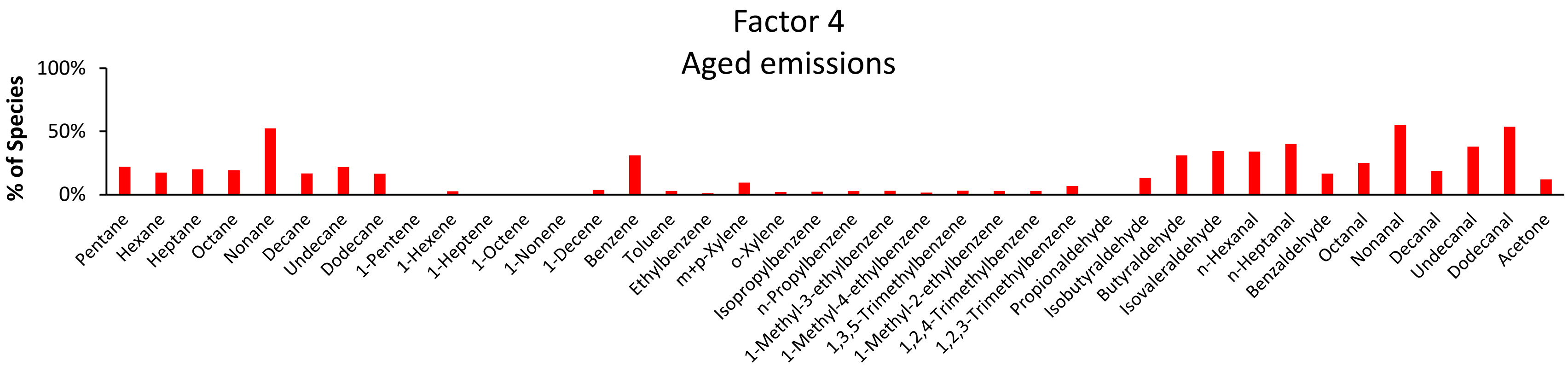

Figure S2: Outdoor PMF-resolved VOC source profiles (% of species)

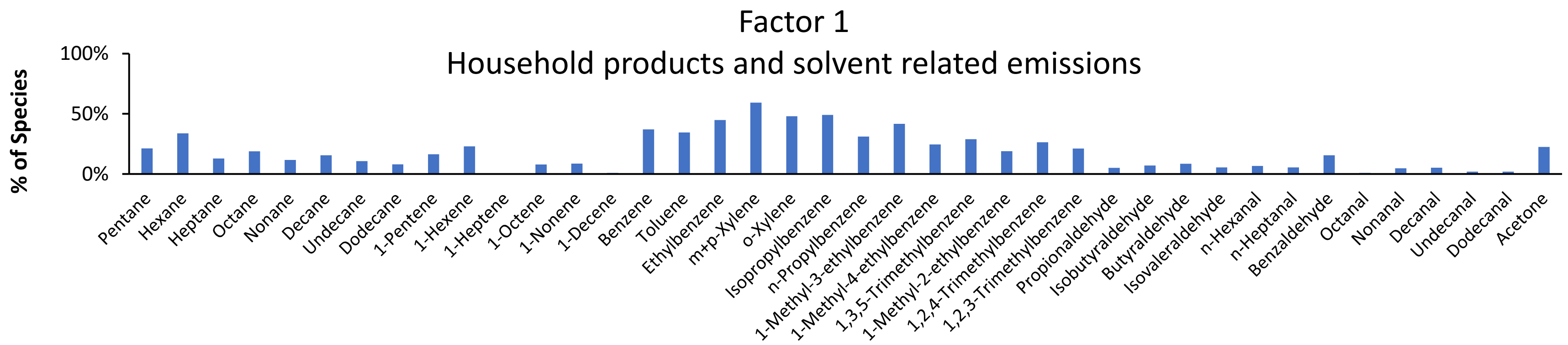
Factor 1
Household products and solvent related emissions
% of Species
100%
50%
0%
Pentane
Hexane
Heptane
Octane
Nonane
Decane
Undecane
Dodecane
1-Pentene
1-Hexene
1-Heptene
1-Octene
1-Nonene
1-Decene
Benzene
Toluene
Ethylbenzene
m+p-Xylene
o-Xylene
Isopropylbenzene
n-Propylbenzene
1-Methyl-3-ethylbenzene
1-Methyl-4-ethylbenzene
1,3,5-Trimethylbenzene
1-Methyl-2-ethylbenzene
1,2,4-Trimethylbenzene
1,2,3-Trimethylbenzene
Propionaldehyde
Isobutyraldehyde
Butyraldehyde
Isovaleraldehyde
n-Hexanal
n-Heptanal
Benzaldehyde
Octanal
Nonanal
Decanal
Undecanal
Dodecanal
Acetone

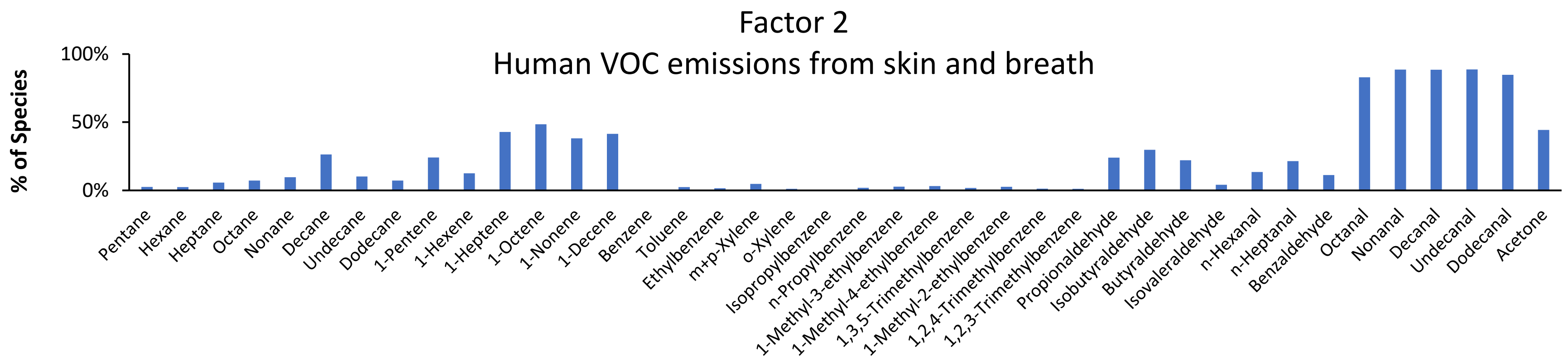
Factor 2
Human VOC emissions from skin and breath
% of Species
100%
50%
0%
Pentane
Hexane
Heptane
Octane
Nonane
Decane
Undecane
Dodecane
1-Pentene
1-Hexene
1-Heptene
1-Octene
1-Nonene
1-Decene
Benzene
Toluene
Ethylbenzene
m+p-Xylene
o-Xylene
Isopropylbenzene
n-Propylbenzene
1-Methyl-3-ethylbenzene
1-Methyl-4-ethylbenzene
1,3,5-Trimethylbenzene
1-Methyl-2-ethylbenzene
1,2,4-Trimethylbenzene
1,2,3-Trimethylbenzene
Propionaldehyde
Isobutyraldehyde
Butyraldehyde
Isovaleraldehyde
n-Hexanal
n-Heptanal
Benzaldehyde
Octanal
Nonanal
Decanal
Undecanal
Dodecanal
Acetone

(continued)

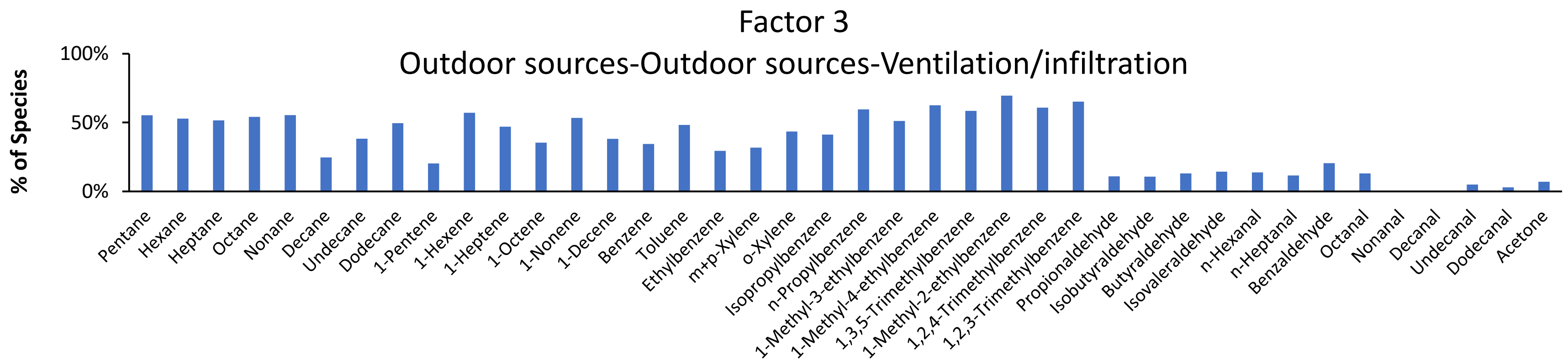

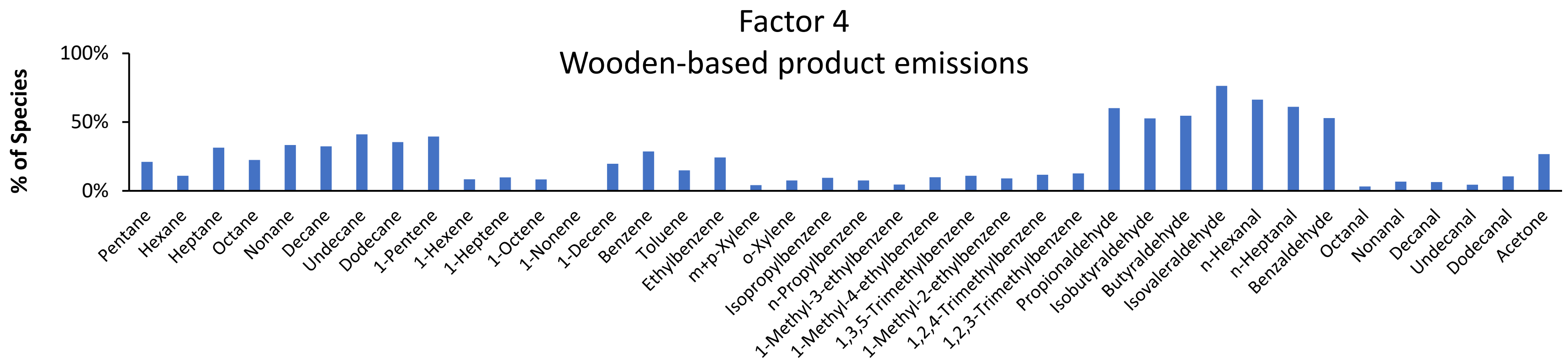

Figure S3: Indoor PMF-resolved VOC source profiles (% of species)

References

1. Besis, A.; Katsaros, T.; Samara, C. Concentrations of Volatile Organic Compounds in Vehicular Cabin Air – Implications to Commuter Exposure. *Environ. Pollut.* **2023**, *330*, 121763, doi:https://doi.org/10.1016/j.envpol.2023.121763.
2. Besis, A.; Latsios, I.; Papakosta, E.; Simeonidis, T.; Kouras, A.; Voliotis, A.; Samara, C. Spatiotemporal Variation of Odor-Active VOCs in Thessaloniki, Greece: Implications for Impacts from Industrial Activities. *Environ. Sci. Pollut. Res. Int.* **2021**, *28*, 59091–59104, doi:10.1007/s11356-020-08573-y.
3. Besis, A.; Georgiadou, E.; Samara, C. Odor-Active Volatile Organic Compounds along the Seafront of Thessaloniki, Greece. Implications for Sources of Nuisance Odor. *Sci. Total Environ.* **2021**, *799*, 149388, doi:https://doi.org/10.1016/j.scitotenv.2021.149388.
